# Global prevalence and antibiotic resistance in clinical isolates of *Stenotrophomonas maltophilia*: a systematic review and meta-analysis

**DOI:** 10.3389/fmed.2023.1163439

**Published:** 2023-05-05

**Authors:** Maryam Banar, Azin Sattari-Maraji, Ghazal Bayatinejad, Elahe Ebrahimi, Leila Jabalameli, Reza Beigverdi, Mohammad Emaneini, Fereshteh Jabalameli

**Affiliations:** ^1^Department of Pathobiology, School of Public Health, Tehran University of Medical Sciences, Tehran, Iran; ^2^Department of Microbiology, School of Medicine, Tehran University of Medical Sciences, Tehran, Iran; ^3^Department of Microbiology, Karaj Branch, Islamic Azad University, Karaj, Iran; ^4^Research Center for Antibiotic Stewardship and Antimicrobial Resistance, Tehran University of Medical Sciences, Tehran, Iran

**Keywords:** *Stenotrophomonas maltophilia*, prevalence, antibiotic resistance, global, meta-analysis

## Abstract

**Introduction:**

*Stenotrophomonas maltophilia* is a little-known environmental opportunistic bacterium that can cause broad-spectrum infections. Despite the importance of this bacterium as an emerging drug-resistant opportunistic pathogen, a comprehensive analysis of its prevalence and resistance to antibiotics has not yet been conducted.

**Methods:**

A systematic search was performed using four electronic databases (MEDLINE via PubMed, Embase, Scopus, and Web of Science) up to October 2019. Out of 6,770 records, 179 were documented in the current meta-analysis according to our inclusion and exclusion criteria, and 95 studies were enrolled in the meta-analysis.

**Results:**

Present analysis revealed that the global pooled prevalence of *S. maltophilia* was 5.3 % [95% CI, 4.1–6.7%], with a higher prevalence in the Western Pacific Region [10.5%; 95% CI, 5.7–18.6%] and a lower prevalence in the American regions [4.3%; 95% CI, 3.2–5.7%]. Based on our meta-analysis, the highest antibiotic resistance rate was against cefuroxime [99.1%; 95% CI, 97.3–99.7%], while the lowest resistance was correlated with minocycline [4·8%; 95% CI, 2.6–8.8%].

**Discussion:**

The results of this study indicated that the prevalence of *S. maltophilia* infections has been increasing over time. A comparison of the antibiotic resistance of *S. maltophilia* before and after 2010 suggested there was an increasing trend in the resistance to some antibiotics, such as tigecycline and ticarcillin-clavulanic acid. However, trimethoprim-sulfamethoxazole is still considered an effective antibiotic for treating *S. maltophilia* infections.

## Introduction

*Stenotrophomonas maltophilia* is an environmental Gram-negative bacillus that has been the subject of extensive research over the last two decades due to its status as the only known species of *Stenotrophomonas* to cause opportunistic infections in humans (1). Before the 1970s, this bacterium was underestimated and was considered a rare opportunistic pathogen with low invasiveness. However, advances in medical interventions and pharmacological treatments have led to an increase in the population of immunocompromised patients, such as those undergoing chemotherapy, organ transplantations, or complex surgeries, who are prone to infection with this bacterium. In addition, the development of diagnostic methods in clinical microbiology resulted in more precise identification of this pathogen. Therefore, the number of reported *S. maltophilia* infections has increased, and it is recognized as an emerging nosocomial pathogen (2). *S. maltophilia* causes infections of the soft tissue, urinary tract, eye, and wound. In addition, it causes pneumonia, bacteremia, sepsis, endocarditis, osteochondritis, mastoiditis, and meningitis (3). Predisposing factors associated with *S. maltophilia* infections include underlying malignancy, indwelling devices, chronic respiratory disease, particularly cystic fibrosis, immune compromisation, prolonged antibiotic use, and long-term hospitalization or admission to an intensive care unit (ICU) (3, 4). The treatment of infections caused by this bacterium presents several challenges. Distinguishing colonization from invasive infections is problematic, and physicians often fail to recognize their associated risk factors and clinical characteristics, which leads to delayed antibiotic prescription and high mortality (5).

Because of the high-level intrinsic resistance of *S. maltophilia* to several classes of antibiotics, there are restricted therapeutic choices for its infections. This bacterium can resist the β-lactam antibiotics (most notably carbapenems) by producing ß-lactamase enzymes, including L1 and L2. It also disrupts the action of aminoglycosides by hydrolyzing enzymes such as acetyl-transferases or modifying the structure of lipopolysaccharide. In addition, low membrane permeability and the overproduction of efflux pumps are other mechanisms that render *S. maltophilia* resistant to a broad range of antibiotics (2, 6). Additionally, they can acquire resistance genes and genetic mutations (7, 8), further limiting the choice of effective antimicrobials. This increasing prevalence of drug-resistant *S. maltophilia* has presented one of the biggest challenges in treating patients in recent years (3, 9).

The Infectious Diseases Society of America (IDSA) has approved a guideline document with recommendations for treating *S. maltophilia* infections (10). Trimethoprim-sulfamethoxazole (TMP/SMX) is the antibiotic of choice for treating these infections, but its use is limited by allergy, intolerance, and increased resistance (11). Other drugs with good susceptibility impact include ticarcillin-clavulanate, ceftazidime, and fluoroquinolones, although resistance to these drugs has been reported. Tetracyclines such as minocycline, tigecycline, and doxycycline are also efficacious in treating *S. maltophilia* infections, and their efficacy has been reported in different geographic areas (3, 12).

The main objective of this study was to assess the global prevalence of *S. maltophilia* and its resistance to commonly used antibiotics. We conducted this systematic review of global human infections due to *S. maltophilia* over the last 31 years.

## Methods

### Search strategy and selection criteria

Four electronic databases, including MEDLINE (via PubMed), Embase, Web of Science, and Scopus, were systematically searched using different combinations of the following keywords: “*Stenotrophomonas maltophilia*” OR “*Xanthomonas maltophilia*” AND “antibiotic resistance” AND “minimum inhibitory concentration” AND “disk agar diffusion” AND “multilocus sequence typing” AND “E-test” AND “antimicrobial resistance gene”. The databases were searched up to 20 October 2019 without any start time limitation.

The study was carried out based on the guidelines of the Preferred Reporting Items for Systematic Reviews and Meta-Analyses (PRISMA) (13). Two distinct reviewers applied the inclusion and exclusion criteria for article selection and screened the titles and abstracts of all studies; then, two autonomous researchers qualified the screened papers. Any disagreements between the reviewers were resolved by consensus.

### Inclusion criteria

Articles were included if they reported the prevalence of *S. maltophilia* isolation among diverse patients in combination with the antibiotic resistance rates of the isolates to various antibiotics, or reported only the antibiotic resistance rates of the isolates. Only articles about the clinical isolates of *S. maltophilia* were enrolled, and studies on the environmental isolates were not considered.

### Exclusion criteria

Conference papers were not evaluated as they did not provide sufficient information for quality assessment. Dissertations and theses were excluded. Articles with unrelated topics, duplicates or overlapping studies, reviews, meta-analyses or systematic reviews, case reports, brief reports, notes, editorials, correspondence, short communications, and letters to the editors were not included. Studies with languages other than English or with unavailable full text were dismissed. Studies that evaluated species other than *S. maltophilia* or tested a total isolate <10 were not assessed. Articles that reported antibiotic resistance as MIC 90 or those that evaluated the combinatorial effects of antibiotics were not enrolled. Studies that considered *S. maltophilia* a Gram-negative bacterium and reported a total antibiotic resistance rate in Gram-negative bacteria were excluded. Articles were removed if they tested only the resistant isolates or reported only the prevalence of *S. maltophilia* infection.

### Study selection and data extraction

Two independent researchers read the included articles in full text and extracted the following details: first author's name, year of study, year of publication, location of the study (country and region), sample size (N/total), type of samples, antibiotic susceptibility testing methods used (agar dilution, broth microdilution, broth macrodilution, E-test, disk agar diffusion [DAD], MIC test strip, Vitek, Phoenix, and Microscan), the antibiotic resistance rate of isolates against various antibiotics, frequency of resistance genes, and frequency of different sequence types. Any discrepancy between the two reviewers was settled by consensus.

### Quality assessment

Two reviewers separately evaluated the quality of the included studies using the Joanna Briggs Institute (JBI) critical appraisal checklist for studies reporting prevalence data (14). This scale rates each criterion out of 1, with a total score ranging from 0 to 10. Studies with a score of ≥5 were classified as high quality.

### Meta-analysis

The meta-analysis was carried out using Comprehensive Meta-Analysis (CMA) software version 2.0 (Biostat, Englewood, NJ). A random-effect model was used for meta-analysis and to pool the estimations. The prevalence of the investigated phenomenon was presented as a forest plot diagram, which shows the estimated prevalence and its relevant 95% confidence interval (CI). Heterogeneity between studies was reported by *I*^2^ statistics. An *I*^2^ between 0 and 25% suggests low heterogeneity, 25–50% indicates moderate heterogeneity, 50–75% represents substantial heterogeneity, and 75–100% shows considerable heterogeneity. Subgroup meta-analysis was employed to compare the prevalence of *S. maltophilia* based on WHO-defined regions and 5-year time intervals. In addition, the antibiotic resistance rates of isolates were compared based on world regions and whether they were reported before or after 2010. To assess the potential risk of publication bias, Begg's rank correlation and Egger's weighted regression methods in combination with a funnel plot were used (*P* < 0.05 was regarded as indicative of a statistically notable publication bias) (15).

## Results

**Figure 1:**
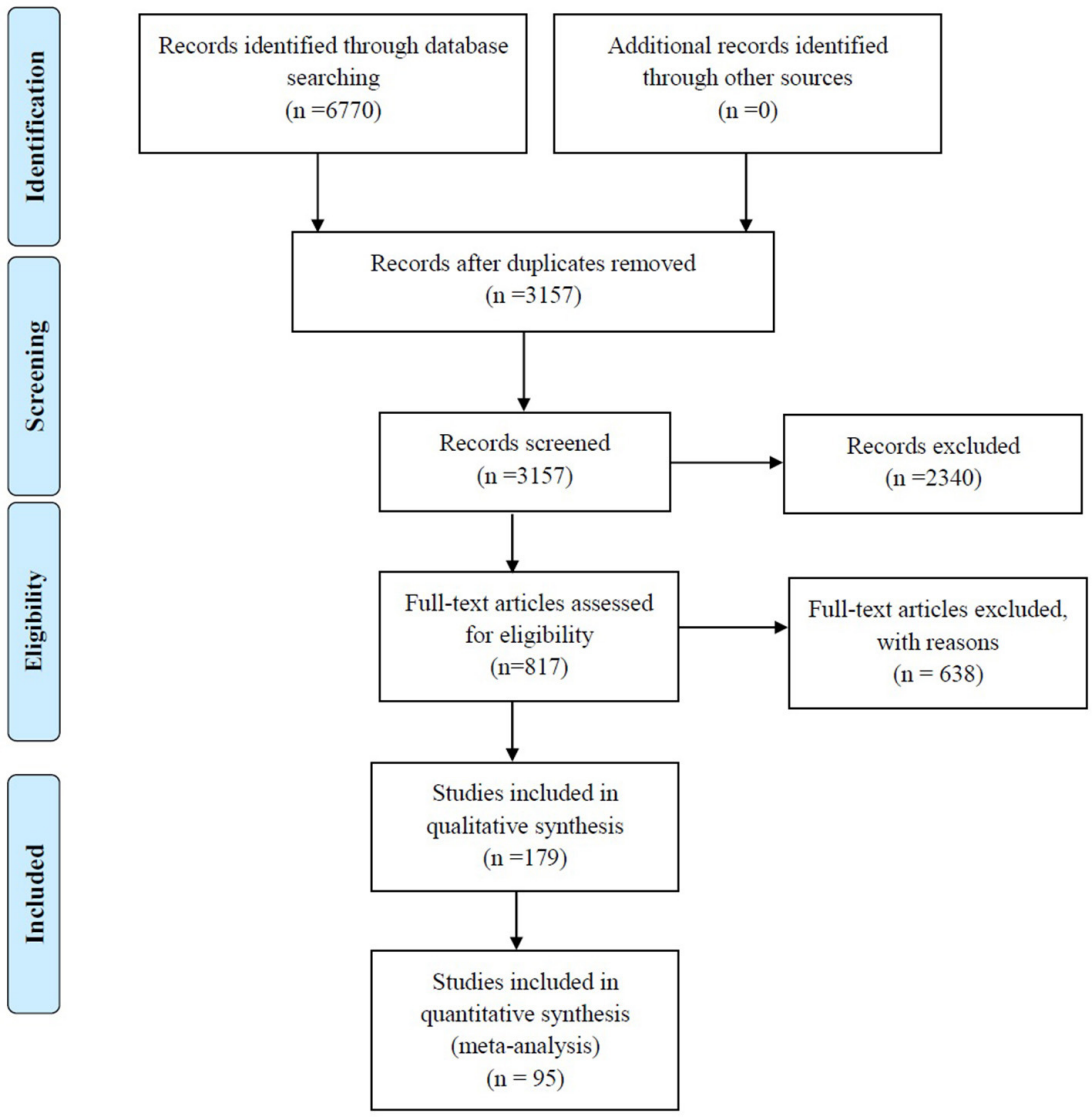
Summary of the literature search and study selection.

A total of 6,770 records were identified through searches of the four aforementioned electronic databases (Figure 1). After removing the 3,613 duplicates, 3,157 unique records were screened based on titles and abstracts, and 2,340 articles were excluded, such as studies with non-relevant topics (*n* = 1,245), repetitive articles (*n* = 470), reviews (*n* = 234), systematic reviews (*n* = 3), case reports (*n* = 64), letters to the editors (*n* = 60), conference abstracts (*n* = 111), editorials (*n* = 9), short surveys (*n* = 10), correspondence (*n* = 2), notes (*n* = 12), reports (*n* = 3), a book (*n* = 1), articles with a total sample of <10 strains (*n* = 17), non-English studies (*n* = 47), and articles that studied environmental samples (*n* = 21). In addition, 30 articles were removed because their full texts were not available. The eligibility of 817 full-text articles was assessed and, ultimately, 179 studies met the inclusion criteria and were enrolled in the qualitative analysis. Of these, 95 studies reporting the prevalence of *S. maltophilia* infection were selected for quantitative analysis (meta-analysis). The characteristics of the 179 included studies are summarized in Table 1.

**Table 1:** Characteristics of the studies that reported *Stenotrophomonas maltophilia* isolation in different parts of the world.

	**References**	**Time of study**	**Time of publication**	**Country**	**WHO regions**	**Type of study**	**Sample size (N/total)**	**Type of samples**	**Patients**	**Quality score**
1	Al-Lawati et al. (16)	ND	2000	Oman	Eastern Mediterranean Region (EMR)	Not-determined (ND)	9/100	Respiratory (7), wound (1), others (1)	Hospitalized patients (ICU)	6
2	Asaad et al. (17)	2012-2013	2013	Saudi Arabia	EMR	Cross-sectional	26/125	Clinical samples	Hospitalized patients	7
3	Bostanghadiri et al. (18)	2016-2017	2019	Iran	EMR	Cross-sectional	164/164	Blood (137), cough swabs (16), nose/throat secretions (9), sputum (1), CSF (1)	Hospitalized patients	4
4	Cunha et al. (19)	1995	1997	Saudi Arabia	EMR	Prospective	27/1132	Clinical samples	Nosocomial infection	6
5	Ebrahim-Saraie et al. (20)	2015-2016	2019	Iran	EMR	Retrospective	44/44	Clinical samples	NICU, ICU, SUR, transplant, general medicine	5
6	El Tahawy and Khalaf (21)	1999-2000	2001	Saudi Arabia	EMR	ND	35/499	Clinical samples	ICU, surgery, pediatric, gynecology	7
7	Jamali et al. (22)	2008-2009	2011	Iran	EMR	ND	100/2300	Blood(100)	Hospitalized patients	7
8	Khalili et al. (23)	2007-2010	2012	Iran	EMR	ND	281/1745	Clinical samples	Hospitalized patients	6
9	Morsi et al. (24)	2013-2015	2016	Egypt	EMR	Cross-sectional	32/32	Urine (1), sputum (7), endotracheal aspirates (15), blood (3), pus (6)	Hospitalized patients	6
10	Qadri et al. (25)	ND	1991	Saudi Arabia	EMR	ND	31/3144	Clinical samples	ND	7
11	Qadri et al. (26)	ND	1992	Saudi Arabia	EMR	ND	28/1205	Clinical samples	ND	7
12	Qadri et al. (27)	ND	1993	Saudi Arabia	EMR	ND	67/1294	Clinical samples	Hospitalized patients	7
13	Qadri et al. (28)	1992	1993	Saudi Arabia	EMR	Cross-sectional	22/563	Clinical samples	Hospitalized patients	7
14	Qadri et al. (29)	ND	1992	Saudi Arabia	EMR	ND	36/922	Clinical samples	Hospitalized patients	7
15	Cha et al. (30)	2006-2014	2016	South Korea	Western Pacific Region (WPR)	Cross-sectional	127/127	Blood (127)	Bacteremia	6
16	Chang et al. (31)	2002	2004	Taiwan	WPR	Cross-sectional	93/93	Sputum (54), wounds (14), central venous catheter (8), urine (5), bile (4), blood (4), throat swabs (2), cerebrospinal fluid (1), eye (1)	ND	5
17	Chen et al. (32)	2002-2006	2010	Taiwan	WPR	Retrospective	67/1307	Blood (67)	Hospitalized patients (hematological malignancy)	7
18	Cho et al. (33)	2009-2014	2015	South Korea	WPR	Retrospective	31/31	Blood (31)	Hospitalized patients (hematological malignancy)	5
19	Cho et al. (34)	2009	2012	South Korea	WPR	ND	33/33	Clinical samples	Hospitalized patients	5
20	Chung et al. (35)	2010	2013	South Korea	WPR	ND	206/206	Clinical samples	ND	5
21	Chung et al. (9)	2009-2010	2015	South Korea	WPR	ND	252/252	Clinical samples	ND	5
22	Fu et al. (36)	ND	2003	China	WPR	ND	323/3905	Clinical samples	ND	7
23	Fujita et al. (37)	1988-1992	1996	Japan	WPR	ND	10/10	Upper respiratory tract (10)	Patients with pneumonia	5
24	Friedman et al. (38)	1988-1997	2002	Australia	WPR	Retrospective	45/45	Blood (45)	Patients with bacteremia	4
25	Hsueh et al. (39)	1999-2003	2005	Taiwan	WPR	ND	451/1006	Clinical samples	ND	6
26	Hu et al. (40)	2006-2008	2011	China	WPR	ND	102/102	Clinical samples	ICU, surgery, oncology, neurology, respiratory care, geriatrics	6
27	Hu et al. (41)	2005-2014	2016	China	WPR	ND	300/300	Clinical samples	ND	6
28	Hu et al. (42)	2010-2011	2014	China	WPR	ND	83/83	Clinical samples	Hospitalized patients	6
29	Hu et al. (43)	2005-2014	2018	China	WPR	ND	300/300	Clinical samples	Hospitalized patients	6
30	Ismail et al. (44)	2011-2012	2017	Malaysia	WPR	ND	84/84	Clinical samples	ND	6
31	Jean et al. (45)	2013-2014	2017	Taiwan	WPR	ND	39/799	Clinical samples	Hospitalized patients	6
32	Kanamori et al. (46)	2009-2010	2015	Japan	WPR	ND	181/181	Clinical samples	Hospitalized patients, community patients	6
33	Liaw et al. (47)	2002-2003	2010	Taiwan	WPR	ND	30/70	Sputum (30)	Sputum, wound, central venous catheter, urine, blood, cerebrospinal fluid, eye	7
34	Liu et al. (48)	2008-2013	2016	Taiwan	WPR	Retrospective	50/378	Blood (50)	Bloodstream infection (BSI)	7
35	Lan et al. (49)	2011-2013	2017	Vietnam	WPR	ND	11/1017	Blood (11)	BSI	7
36	Neela et al. (50)	2008	2012	Malaysia	WPR	ND	64/64	Tracheal aspirate (25), peritoneal fluid (1), bronchoalveolar lavage (1)	ICU, neurology, psychiatric, dermatology wards	6
37	Ning et al. (51)	2007-2011	2013	China	WPR	ND	17/127	Sputum (17)	Patients with VAP in a pediatric ICU	6
38	Rhee et al. (52)	2007-2011	2013	South Korea	WPR	ND	121/121	Clinical samples	ND	6
39	Shi et al. (53)	2003-2006	2009	China	WPR	Cross-sectional	48/323	Blood (48)	Hospitalized (liver transplant)	7
40	Sun et al. (54)	2006-2012	2016	China	WPR	Cross-sectional	51/51	Pus (7), intravascular catheter (7), postoperative and burn wound (7), bronchial secretions/lavage (6), urinary catheter (6), urine (5), sputum (4), bile (4), blood (3), ascitic fluid (2)	Hospitalized patients with invasive infections	6
41	Tan et al. (55)	2004	2006	Singapore	WPR	Cross-sectional	17/ 102	Clinical samples	ND	7
42	Tanimoto et al. (56)	2005	2013	Japan	WPR	ND	66/66	Clinical samples	ND	6
43	Wang et al. (57)	1998	2000	China	WPR	Cross-sectional	50/440	Clinical samples	ND	7
44	Wang et al. (58)	1999-2003	2004	Taiwan	WPR	Cross-sectional	50/50	Blood (50)	Hospitalized patients (bacteremia)	6
45	Wei et al. (59)	2013	2016	China	WPR	Cross-sectional	80/80	Respiratory tract specimens (63), catheter-related specimens (10), urine (4), blood (3)	ND	6
46	Wu et al. (60)	1998-2008	2012	Taiwan	WPR	Cross-sectional	377/377	Respiratory tract (256), blood (48), others (73)	Hospitalized (ICU)/outpatient patients (60)	6
47	Watanabe et al. (61)	1994-2011	2014	Australia	WPR	Comparative analysis	40/40	Clinical samples	ND	6
48	Xu et al. (62)	2005-2008	2010	China	WPR	ND	12/258	Clinical samples	Neonate patients (NICU)	7
49	Yuk-Fong Liu et al. (63)	1993-1994	1995	Taiwan	WPR	ND	28/366	Clinical samples	Hospitalized patients (ICU)	7
50	Zhao et al. (64)	2015	2017	China	WPR	Cross-sectional	400/400	Sputum (315), throat swab (30), urine (25), secretions (15), bile (10), blood (5)	Hospitalized patients	5
51	Zhao et al. (65)	2012-2014	2016	China	WPR	Cross-sectional	450/450	Clinical samples	Hospitalized patients	6
52	Zhao et al. (66)	2012-2015	2018	China	WPR	Cross-sectional	450/450	Respiratory tract specimens (450)	Hospitalized patients	6
53	Zhang et al. (67)	ND	2012	China	WPR	Cross-sectional	442/442	Clinical samples	ND	6
54	Chawla et al. (68)	2009-2011	2013	India	South-East Asia Region (SEAR)	Retrospective	15/33	Respiratory samples (15)	Respiratory tract infection	7
55	Chawla et al. (69)	2012-2013	2014	India	SEAR	Retrospective	33/33	Sputum (17), endotracheal aspirates (16)	Patients with lower respiratory tract infection (LRTI)	6
56	Garg et al. (70)	2014-2016	2019	India	SEAR	ND	5/3414	Clinical samples	ND	5
57	Gunasekar et al. (71)	2017	2018	India	SEAR	ND	12/240	ND	ND	7
58	Kaur et al. (72)	2012-2013	2015	India	SEAR	ND	106/106	Clinical samples	Hospitalized patients	6
59	Nayyar et al. (73)	2015-2016	2017	India	SEAR	Retrospective	23/2734	Blood (15), urine (4), tracheal aspirate (4)	Pediatric patients	6
60	Paopradit et al. (74)	2014-2015	2017	Thailand	SEAR	ND	64/64	Sputum (36), blood (9), tissue (6), pus (1), urine (1), body fluid (9), bronchial wash (2)	Patients on the ICU, respiratory care unit (RCU), medicine (MED), surgical, pediatric, emergency room, eye wards	6
61	Tantisiriwat et al. (75)	2014-2015	2017	Thailand	SEAR	Cross-sectional	33/ 1288	Sputum, urine, pus, blood	ND	6
62	Averbuch et al. (76)	2001-2014	2017	Israel	European Region (EUR)	Retrospective	10/116	Blood (10)	Hospitalized children (malignancies and solid tumors)	7
63	Averbuch et al. (77)	2014-2015	2017	Israel	EUR	Non-interventional prospective	31/704	Blood (31)	Patients with hematopoietic stem cell transplant (HSCT)	7
64	Bousquet et al. (78)	2003-2010	2014	France	EUR	Retrospective	45/723	Blood (45)	Hematological malignancies	5
65	Canton et al. (79)	1991- 1998	2002	Spain	EUR	ND	98/127	Respiratory secretion, Sputum	Hospitalized patients (CF and non-CF)	5
66	Chen et al. (80)	1991	1993	UK, Ireland	EUR	ND	21/6724	Clinical materials except feces	Hospitalized patients	6
67	De Dios Caballero et al. (81)	2013	2015	Spain	EUR	Prospective, multicenter, observational	49/339	Sputum (49)	CF patients	7
68	Cikman et al. (82)	2006-2012	2016	Turkey	EUR	Retrospective	118/118	Tracheal aspirate (67), blood (17), sputum (10), wound (10), ear (3), CSF (2), paracentesis (2), pleural fluid (2), urine (2), puncture fluid (2), catheter (1)	ND	5
69	Di Bonaventuraa et al. (83)	2001	2002	Italy	EUR	ND	19/223	Respiratory tract specimen, blood, urine, skin and wound swabs	Neutropenic patients with hematological malignancies	6
70	Di Bonaventuraa et al. (84)	ND	2004	Italy	EUR	ND	50/50	Clinical samples	Neutropenic patients with hematological malignancies	6
71	Djordjevic et al. (85)	2009-2015	2017	Serbia	EUR	Cohort	38/850	Sputum, BAL, tracheal samples	Medical-Surgical ICU/HAP and VAP	7
72	Esposito et al. (86)	2003-2014	2017	Italy	EUR	ND	91/91	Sputum samples (91)	CF patients	5
73	Frank et al. (87)	1996-1997	2000	Germany	EUR	ND	52/52	Tracheal secretions, wound, blood, urine, biopsy, puncture fluid	ND	6
74	Fadda et al. (88)	1997-1999	2004	Italy	EUR	ND	307/307	Respiratory tract samples (307)	Hospitalized patients	6
75	Gajdacs et al. (2)	2008-2017	2019	Hungary	EUR	Retrospective	579/579	Tracheal aspirates, sputum, BAL, pleural and pericardial puncture	Septicemia, hematological malignancies and solid tumors, pneumonia, pleuritis, CF, meningitis, etc.	4
76	Galani et al. (89)	2004-2006	2008	Greece	EUR	ND	36/778	Clinical samples	ND	6
77	Garcia-Rodriguez et al. (90)	1992	1995	Spain	EUR	ND	21/2426	Clinical samples	ND	6
78	Garcia-Rodriguez et al. (91)	1991	1989	Spain	EUR	ND	42/42	Clinical samples	ND	5
79	Garcia-Rodriguez et al. (92)	ND	1991	Spain	EUR	ND	18/18	Clinical samples	ND	5
80	Gesu et al. (93)	2000	2003	Italy	EUR	ND	124/4003	Clinical samples	ND	6
81	Glupczynski et al. (94)	1996-1997	2001	Belgium	EUR	ND	73/73	Clinical samples	ICU patients	6
82	Glupczynski et al. (94)	1998-1999	2001	Belgium	EUR	ND	48/48	Clinical samples	ICU patients	6
83	Gómez-Garces et al. (95)	1996-2006	2009	Spain	EUR	ND	80/228	Clinical samples	ND	7
84	Goncalves-Vidigal et al. (96)	2009-2011	2011	Germany	EUR	ND	65/65	Sputum (65)	CF patients	6
85	Gordon et al. (97)	ND	2010	UK	EUR	ND	13/13	Sputum, blood	ND	4
86	Gospodarek et al. (98)	1994-1995	1997	Poland	EUR	ND	27/27	Wound smears, pus, intubation tube	Intensive therapy, urologic, neurology, surgery	5
87	Gramegna et al. (99)	2001-2010	2018	UK	EUR	ND	34/193	Sputum (34)	CF patients	7
88	Grillon et al. (100)	ND	2016	France	EUR	ND	40/120	Clinical samples	ND	7
89	Grohs et al. (101)	ND	2017	France	EUR	ND	12/58	Respiratory samples (12)	CF patients	7
90	Guembe et al. (102)	2003-2007	2008	Spain	EUR	ND	7/572	Wound, abscesses	Patients with intra-abdominal infection	7
91	Gulmez et al. (103)	2005	2010	Turkey	EUR	ND	25/25	Clinical samples	Hospitalized patients	5
92	Gulmez et al. (104)	1998-2003	2005	Turkey	EUR	ND	205/205	Clinical samples	Hospitalized patients	6
93	Guriz et al. (105)	1995-2005	2008	Turkey	EUR	ND	33/33	Blood (33)	Hospital-acquired bacteremia	6
94	Hohl et al. (106)	ND	1991	Switzerland	EUR	ND	33/33	Clinical samples	ND	6
95	Hombach et al. (107)	2010-2011	2012	Germany	EUR	ND	160/3713	Clinical samples	ND	7
96	Hoban et al. (108)	1997-1999	2001	16 European countries	EUR	ND	578/21464	Clinical samples	ND	7
97	Juhász et al. (109)	2009-2011	2014	Hungary	EUR	ND	100/160	Clinical samples	Hospitalized patients	7
98	Klietmann et al. (110)	1986-1989	1991	Germany	EUR	ND	234/130033	Clinical samples	ND	7
99	Koseoglu et al. (111)	1998-2001	2014	Turkey	EUR	ND	40/40	Clinical samples	Pediatric patients	6
100	Kucukates et al. (112)	2000-2002	2005	Turkey	EUR	ND	16/367	Clinical samples	Hospitalized patients (coronary and surgical ICUs)	7
101	Lakatos et al. (113)	1993-2013	2014	Switzerland	EUR	ND	27/27	Blood (27)	Bacteremia	4
102	Lanzafame et al. (114)	ND	2005	Italy	EUR	ND	64/495	ND	Patients hospitalized in intensive care, onco-hematological, surgical, burn and transplant units	7
103	Livermore et al. (115)	1991 and 2001	2003	UK, Ireland	EUR	ND	23/5031	Clinical samples	Hospitalized patients	6
104	Livermore et al. (116)	2008-2012	2014	UK	EUR	ND	40/170	ND	CF patients	7
105	Madi et al. (117)	2013-2015	2016	Serbia	EUR	Retrospective	88/88	Clinical samples	CF, non-CF outpatients and inpatients	6
106	McKnight et al. (118)	ND	2005	Ireland	EUR	ND	10/60	Sputum (10)	CF patients	7
107	Micozzi et al. (119)	1987-1996	2000	Italy	EUR	Retrospective	26/26	Blood (26)	Patients with hematologic malignancies (bacteremia)	5
108	Milne et al. (120)	2001-2010	2012	UK	EUR	ND	80/80	Respiratory samples (80)	CF patients	6
109	Pasargiklian et al. (121)	1993	1996	Italy	EUR	ND	25/303	Broncho aspirate (25)	ICU patients	7
110	Samonis et al. (122)	2005-2010	2012	Greece	EUR	Retrospective	68/81	Bronchial secretions/lavage (23), sputum (15), pus (8), blood (7), intravascular catheter tip (4), urine (4), ascitic fluid (3), bile (3), contact lenses (3), cornea (1), peritoneal dialysis fluid (1), throat swab (1), bone (1)	Hospitalized/outpatient patients (5.9%)	7
111	Samonis et al. (123)	2008	2010	Greece	EUR	Retrospective	21/594	Blood, lower respiratory tract, pus, normally sterile fluids, central venous catheter tips, stool, ophthalmic specimens, upper respiratory tract, genital tract	Hospitalized/outpatient patients (10.3%)	7
112	Schmitz et al. (124)	1997-1998	1999	Austria, Belgium, France, Germany, Greece, Italy, Netherlands, Poland, Portugal, Spain, Switzerland	EUR	Cross-sectional	106/9682	Blood, respiratory tract, wound, urine	ND	7
113	Traub et al. (125)	ND	1987	Germany	EUR	ND	14/14	Clinical samples	ND	4
114	Traub et al. (126)	1986-1997	1998	Germany	EUR	ND	96/96	Clinical samples	ICU patients	6
115	Tripodi et al. (127)	ND	2001	Italy	EUR	ND	50/50	Clinical samples	ND	6
116	Tunger et al. (128)	2003-2005	2007	Turkey	EUR	Retrospective	35/35	Blood (35)	Hospitalized patients (bacteremia)	6
117	Usarek et al. (129)	2011-2014	2016	Poland	EUR	Retrospective	26/26	Blood (26)	Hospitalized patients (blood infection)	4
118	Valenza et al. (130)	2006	2008	Germany	EUR	Cross-sectional	70/464	Sputum (70)	CF patients	7
119	Adams-Sapper et al. (131)	2007-2009	2012	USA	Region of the Americas (AMR)	Cross-sectional	9/376	Blood (9)	Hospitalized patients, outpatients, jail clinics (bloodstream infection)	6
120	Alcaraz et al. (132)	2004-2012	2018	Argentina	AMR	Cross-sectional	63/63	Respiratory specimens, blood, renal biopsy, peritoneal fluids, urine	Non-CF patients exposed to invasive devices	5
121	Blondeau et al. (133)	1994-1995	1999	Canada	AMR	ND	31/1518	Clinical samples	ND	7
122	Church et al. (134)	1999-2009	2012	Canada, USA	AMR	ND	90/90	Blood (62), lower respiratory tract specimen (19), peritoneal fluid (5), cerebrospinal fluid (4)	Hospitalized patients (invasive infections)	6
123	Denisuik et al. (135)	2007-2016	2018	Canada	AMR	National surveillance	238/8130	Respiratory specimen, blood, wound, urine	Patients with respiratory infections, urine, wound and BSIs.	7
124	Flamm et al. (136)	2015	2019	USA	AMR	ND	102/2254	Clinical samples	ND	7
125	Flores-Treviño et al. (137)	2006-2013	2014	Mexico	AMR	ND	119/119	Respiratory tract, blood, wound	ICU Patients	6
126	Forrester et al. (138)	ND	2018	USA	AMR	ND	13/93	Respiratory specimens (13)	CF patients	7
127	Fuchs et al. (139)	1994	1996	USA	AMR	ND	74/74	Clinical samples	ND	6
128	Gerlach et al. (140)	ND	1992	USA	AMR	ND	76/3416	Clinical samples	ND	7
129	Herrera-Heredia et al. (141)	2007-2015	2017	Mexico	AMR	ND	196/196	Clinical samples	ND	6
130	Hoban et al. (142)	1997-1999	2003	Canada, USA	AMR	ND	110/4536	Clinical samples	ND	7
131	Isenberg et al. (143)	1996-1997	1999	USA	AMR	ND	20/60	Clinical samples	ND	7
132	Jones et al. (144)	1995-1996	1997	USA	AMR	ND	18/270	Blood (18)	Nosocomial BSI	7
133	Jones et al. (145)	1997	1999	Canada, USA, Latin America	AMR	ND	177/23000	Clinical samples	ND	7
134	Karlowsky et al. (146)	2010-2012	2013	Canada	AMR	ND	174/9758	Clinical samples	ND	7
135	Karlowsky et al. (147)	2009-2009	2011	Canada	AMR	ND	79/4546	Clinical samples	ND	7
136	Karlowsky et al. (148)	2000-2000	2002	USA	AMR	ND	94/3099	Clinical samples	ND	7
137	Krueger et al. (149)	ND	2001	USA	AMR	ND	23/23	Urine, sputum, wound	ND	5
138	Mutnick et al. (150)	2000-2001	2013	USA	AMR	ND	54/1992	ND	Hospitalized patients in the oncology center (bloodstream, respiratory, urinary, skin and soft tissues infections)	7
139	Nicodemo et al. (151)	2000-2002	2004	Brazil	AMR	ND	70/70	Respiratory (47), urine (6), biopsy tissues (4), blood (3) and others (10)	Hospitalized patients	6
140	Passerini De Rossi et al. (152)	2004-2008	2009	Argentina	AMR	ND	32/32	Clinical samples	Patients with device-associated nosocomial infection	6
141	Poulos et al. (153)	ND	1995	Canada, USA	AMR	ND	31/31	Clinical samples	ND	5
142	Rizek et al. (154)	ND	2015	Brazil	AMR	ND	48/153	Blood (48)	ND	7
143	Rolston et al. (155)	ND	2003	USA	AMR	Cross-sectional	40/924	Clinical samples	Hospitalized patients (cancer patients)	7
144	Rolston et al. (156)	ND	1997	USA	AMR	Cross-sectional	30/716	Clinical samples	Hospitalized patients (cancer patients)	7
145	Rutter et al. (157)	2010-2014	2016	USA	AMR	Cross-sectional	45/542	Respiratory samples (45)	Hospitalized patients (CF patients)	7
146	Sader et al. (158)	2015-2017	2018	USA	AMR	Cross-sectional	311/6091	Trans tracheal aspiration, bronchoalveolar lavage, protected brush samples, qualified sputum samples	Hospitalized patients (pneumonia patients)	7
147	Sader et al. (159)	ND	1993	USA	AMR	ND	10/853	Clinical samples	Hospitalized patients (septicemia)	6
148	Sahm et al. (160)	1999	2001	USA	AMR	Cross-sectional	123/3368	Clinical samples	ND	7
149	San Gabriel et al. (161)	1996- 2001	2004	USA	AMR	Cross-sectional	955/955	Respiratory samples (955)	CF patients	6
150	Sattler et al. (162)	1992-1998	2000	USA	AMR	Retrospective	51/51	Blood (32), conjunctiva (3), urine (3), skin and soft tissue (3), surgical site or wound (3), paranasal sinus (3), other sites (4)	ND	6
151	Travassos et al. (163)	ND	2004	Brazil	AMR	ND	39/39	ND	Hospitalized/outpatient patients (9)	6
152	Spierer et al. (164)	2000–2013	2018	USA	AMR	Retrospective	15/58	Corneal (15)	Keratitis patients	7
153	Zhanel et al. (165)	2007-2009	2011	Canada	AMR	Cross-sectional	245/18538	Blood, urinary tract, respiratory tract, wound	Inpatients and outpatients	7
154	Zhanel et al. (166)	2014–2015	2018	Canada	AMR	Cross-sectional	118/4637	Blood, urinary tract, respiratory tract, wound	ND	7
155	Zhanel et al. (167)	2005-2006	2008	Canada	AMR	Cross-sectional	108/3931	Blood, urine, wound/tissue, respiratory tract	Hospitalized patients (ICU)	7
156	Chow et al. (168)	2002	2006	China, Taiwan, Korea, Australia, Thailand, Malaysia, USA, Spain, Germany, Belgium, Italy, Mexico, Puerto Rico, Guatemala, Argentina, Ecuador, Venezuela	Multiple regions	Prospective	36/3134	ND	Patients with intra-abdominal infections	7
157	Corlouer et al. (169)	2013-2014	2017	France, Spain, Tunisia	Multiple regions	Collection study	83/83	Sputum (16), tracheal aspiration (10), protected distal specimen (7), bronchoalveolar lavage (2), blood (18), urine (9), suppuration (8), central arterial/venous catheter (4), others (9)	CF patients, solid cancer, hematological malignancy and organ transplant	5
158	Diez-Aguilar et al. (170)	2003-2016	2019	Netherlands, Ireland, Spain, USA, Australia	Multiple regions	Cross-sectional	106/286	Respiratory samples (106)	CF patients	7
159	Farrell et al. (171)	2005-2010	2014	Europe, Israel, Turkey	Multiple regions	ND	420/60084	Clinical samples	Hospitalized patients	7
160	Farrell et al. (172)	2003-2008	2010	Asia-pacific, Europe, Latin America, North America	Multiple regions	ND	1586/1586	Clinical samples	Bloodstream and respiratory tract infections	6
161	Fedler et al. (173)	2004	2006	North America, Latin America, Europe	Multiple regions	ND	53/3537	Clinical samples	Pediatric patients	7
162	Flamm et al. (174)	2013	2016	USA, Europe-Mediterranean, Latin America, Asia-pacific	Multiple regions	ND	464/464	Clinical samples	ND	6
163	Frei et al. (175)	ND	1994	USA, Canada, Brazil, Japan, Spain, Switzerland	Multiple regions	ND	61/61	Clinical samples	ND	6
164	Fritsche et al. (176)	2000-2004	2005	Asia, Australia, Europe, North America, South America	Multiple regions	ND	57/10763	ND	Patients with community-acquired respiratory tract infections	7
165	Gales et al. (2001b)	1997-1999	2001	Asia-pacific, Europe, Latin America, Canada, USA	Multiple regions	The SENTRY Antimicrobial Surveillance Program	842/70067	Blood, Respiratory, wound, urine	BSIs (objective A), pneumonia in hospitalized patients (objective C), skin/soft-tissue infections (objective D), and urinary tract infections (objective E)	7
166	Gales et al. (177)	2001-2004	2006	Asia-pacific, Europe, Latin America, Canada, USA	Multiple regions	ND	1256/13808	Clinical samples	ND	7
167	Gales et al. (178)	2002-2005	2008	Asia-pacific, Europe, Latin America, Canada, USA	Multiple regions	ND	763/763	Blood, respiratory tract samples	ND	6
168	Hoban et al. (179)	ND	1993	6 countries	Multiple regions	ND	61/6064	Clinical samples	ND	7
169	Jones et al. (180)	1997-2001	2003	Asia-pacific, Europe, Latin America, US, Canada	Multiple regions	ND	1488/18569	Clinical samples	ND	7
170	Liu et al. (181)	2003-2010	2012	Taiwan, Thailand, Vietnam, Philippines, Hong Kong, China, Malaysia, Singapore, South Korea, Australia, New zealand	Multiple regions	Prospective	204/20710	Tissue, wound, fluid obtained from paracentesis or percutaneous aspiration of abscesses	Patients with intra-abdominal infections (IAI)	7
171	Renteria et al. (182)	2007-2012	2014	Egypt, Morocco, Mauritius, Namibia, South Africa, Tunisia, Israel, Jordan, Lebanon, Oman, Saudi Arabia	Multiple regions	ND	16/2245	Body fluids, stomach, large and small colon, rectum, liver, gall bladder, pancreas, other intra-abdominal organs	Hospitalized patients	7
172	Sader et al. (183)	2011-2014	2016	Argentina, Brazil, Chile, Colombia, Costa Rica, Ecuador, Guatemala, Mexico, Panama, Peru, Venezuela	Multiple regions	Cross-sectional	141/13494	Clinical samples	ND	7
173	Sader et al. (184)	2009–2012	2014	USA, Belgium, France, Germany, Greece, Ireland, Italy, Poland, Portugal, Spain, Sweden, UK, Turkey, Israel	Multiple regions	Cross-sectional	330/8201	Trans tracheal aspiration, bronchoalveolar lavage, protected brush samples, qualified sputum samples	Hospitalized patients (Pneumonia patients)	7
174	Sader et al. (185)	2011	2013	USA, Canada, Belgium, Czech Republic, France, Germany, Greece, Ireland, Israel, Italy, Poland, Portugal, Romania, Russia, Slovakia, Slovenia, Spain, Sweden, Turkey, United Kingdom, Ukraine, Argentina, Brazil, Chile, Mexico, Australia, China, Hong Kong, India, Japan, Korea, Malaysia, New Zealand, Singapore, Taiwan, Thailand	Multiple regions	Cross-sectional	362/362	Clinical samples	Hospitalized patients (BSI, respiratory tract infections, wound and skin infections)	7
175	Sader et al. (186)	2000–2004	2005	ND	Multiple regions	Cross-sectional	131/9093	Clinical samples	Hospitalized patients (ICU)	7
176	Thomson et al. (187)	ND	1999	USA, Czech Republic, Hungary, Spain, Sweden, the United Kingdom, Australia	Multiple regions	ND	16/296	ND	ND	6
177	Toleman et al. (188)	1998–2003	2007	ND	Multiple regions	Cross-sectional	1744/1744	ND	ND	6
178	Tsiodras et al. (189)	1993-1997	2000	USA, Switzerland	Multiple regions	Retrospective case series	69/1279	Clinical samples	Hospitalized patients	7
179	Yamane et al. (190)	1992	1994	USA, Canada, Brazil, Japan, Switzerland, Spain	Multiple regions	Cross-sectional	61/889	Clinical samples	ND	7

Overall, 179 studies conducted during the 31-year period between 1986 and 2017 were included. The articles had a wide geographical distribution, and the studies featured in them were carried out in different parts of the world. According to the World Health Organization's (WHO) regions, most studies were from the European Region (*n* = 57, 32%), followed by the West-Pacific Region (*n* = 39, 22%), the Region of the Americas (*n* = 37, 21%), the Eastern Mediterranean Region (*n* = 14, 8%), and the South-East Asian Region (*n* = 8, 4%). There was no independent study from the African Region. Twenty-four studies (13%) were conducted across different continents and were, therefore, classified as multiple region studies and did not conform to the WHO categories (Table 1).

The studies had very different sample sizes, ranging from 10 to 130,033. A total of 580,963 samples were examined, of which 25,596 were positive for *S. maltophilia*. Of the 179 studies, only 58 reported the types and details of examined specimens (5,106 samples). The most frequent sources of *S. maltophilia* isolation were respiratory samples (*n* = 3,434, 67%) and blood (*n* = 1,223, 24%) (Table 1). The qualities of all the reviewed studies were evaluated using the JBI critical appraisal checklist. Of the 95 studies included in the meta-analysis, 78 (82%) scored seven, 16 (17%) scored six, and one (1%) scored five. Therefore, all the studies enrolled in the meta-analysis had a high-quality score (a score of five or more) (Table 1).

### Prevalence of *Stenotrophomonas maltophilia* by WHO regional offices

**Figure 2:**
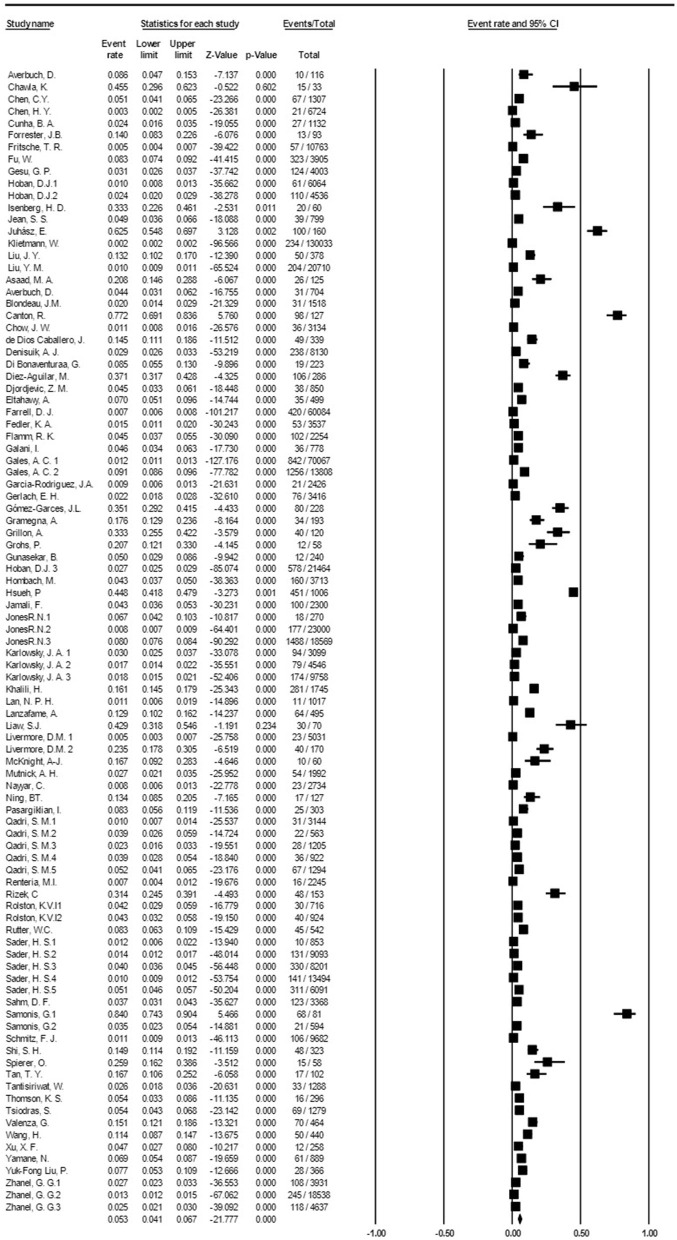
Forest plot diagram of the global prevalence rate of *S. maltophilia* isolation from clinical samples. The middle point of each line indicates the prevalence rate, and the length of the line indicates the 95% confidence interval of each study.

Based on the meta-analysis, the pooled prevalence rate of global *S. maltophilia* infection was estimated to be 5.3 % [95% CI, 4.1–6.7%] (Table 2 and Figure 2). Egger's test did not demonstrate publication bias (*P* > 0.05). However, Begg's test showed evidence of publication bias in the 95 analyzed studies (*P* = 0.017). Additionally, the corresponding funnel plot indicated publication bias (Supplementary File 1). Results demonstrated high heterogeneity (*I*^2^= 99.428%; *P* = 0.000) among the selected studies (Table 2).

**Table 2:** Meta-analysis of the global prevalence rate of *Stenotrophomonas maltophilia* isolation from clinical samples.

	**No. of studies**	**Prevalence of *S. maltophilia* isolation [95% CI]**	**N/total**	**Heterogeneity test, *I^2^***	**Heterogeneity test, *P*-value**	**Begg's test**	**Egger's test**
Overall	95	5.3 [4.1–6.7]	11557/561463	99.428	0.000	0.017	0.367

**Figure 3:**
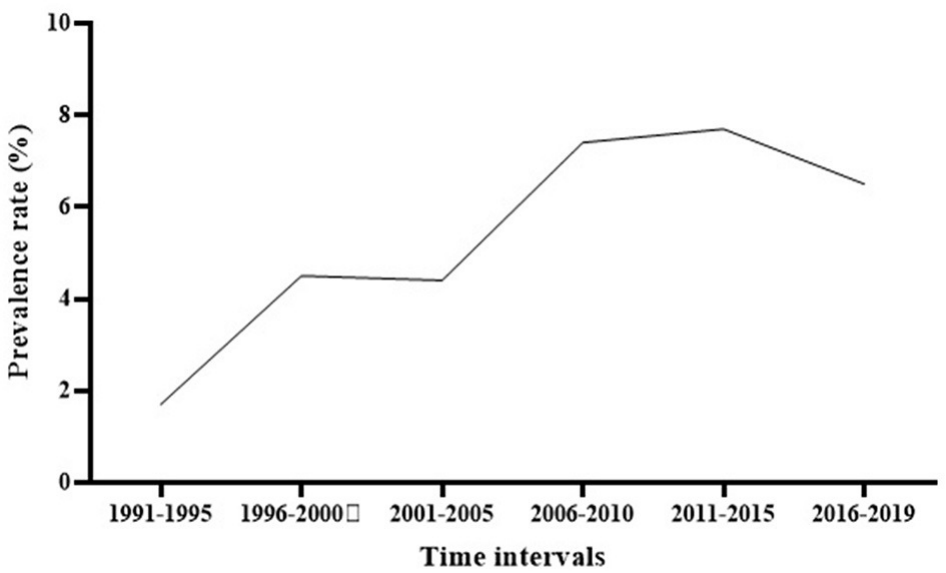
The global prevalence of *S. maltophilia* isolation based on the publication time of studies.

Subgroup meta-analysis based on the publication period of the studies (from 1991 to 2019) revealed that the prevalence rate of *S. maltophilia* isolation had an increasing trend over time, from 1.7% [95% CI, 0.7–4%] between 1991 and 1995 to 6.5% [95% CI, 4.1–10.1%] between 2016 and 2019. The highest prevalence rate [7.7%; 95% CI, 4.3–13.4 %] was observed between 2011 and 2015 (See Figure 3 and Table 3) (Supplementary File 1).

**Table 3:** Subgroup meta-analysis of the global prevalence rate of *Stenotrophomonas maltophilia* isolation from clinical samples.

**Subgroups**		**No. of studies**	**Prevalence of *S. maltophilia* isolation [95% CI]**	**N/total**	**Heterogeneity test, *I^2^***	**Heterogeneity test, *P*-value**	**Begg's test**	**Egger's test**
Time of publication	1991-1995	13	1.7 [0.7–4.0]	696/157899	99.155	0.000	0.502	0.036
	1996-2000	11	4.5 [2.2–8.8]	569/38696	98.493	0.000	0.119	0.003
	2001-2005	17	4.4 [2.7–7.1]	4159/156226	99.525	0.000	0.232	0.983
	2006-2010	13	7.4 [4.5–12.1]	1834/28534	98.529	0.000	0.951	0.620
	2011-2015	20	7.7 [4.3–13.4]	2465/135819	99.517	0.000	0.047	0.011
	2016-2019	20	6.5 [4.1–10.1]	1383/43283	98.555	0.000	0.381	0.157
World regions	Asia (Total)	27	7.1 [4.6–10.7]	1879/27322	98.71	0.000	0.738	0.025
	Asia (EMR)^*^	10	4.7 [2.6–8.6]	653/12929	98.146	0.000	0.858	0.035
	Asia (SEAR)	4	5.2 [1.1–20.9]	83/4295	97.709	0.000	0.308	0.237
	Asia (WPR)	13	10.5 [5.7–18.6]	1143/10098	98.823	0.000	0.760	0.301
	EUR	29	7.9 [4.3–14]	2173/190229	99.453	0.000	0.586	0.008
	AMR	26	4.3 [3.2–5.7]	2593/105324	98	0.000	0.0325	0.0148

^*^EMR, Eastern Mediterranean Region; SEAR, South-East Asia Region; WPR, Western Pacific Region; EUR, European Region; AMR, Regions of the Americas.

**Figure 4:**
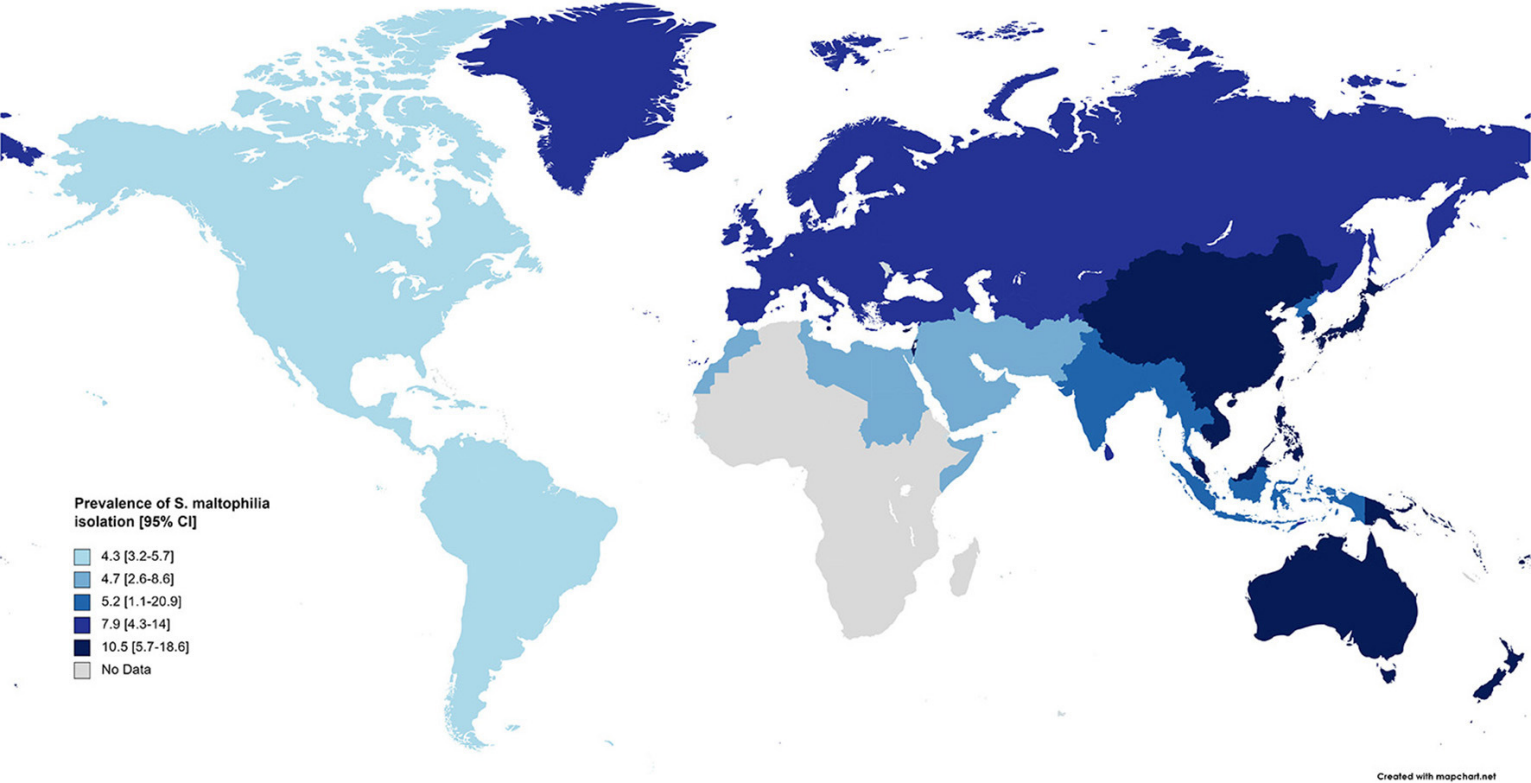
Prevalence of *S. maltophilia* isolated from clinical samples, by WHO regions.

Subgroup meta-analysis based on the world regions defined by WHO revealed that the highest prevalence of *S. maltophilia* infections occurred in the Western Pacific Region [10.5%; 95% CI, 5.7–18.6%] and the European Region [7.9%; 95% CI, 4.3–14%]. The lowest prevalence occurred in the Region of the Americas [4.3%; 95% CI, 3.2–5.7%] (see Table 3 and Figure 4).

**Figure 5:**
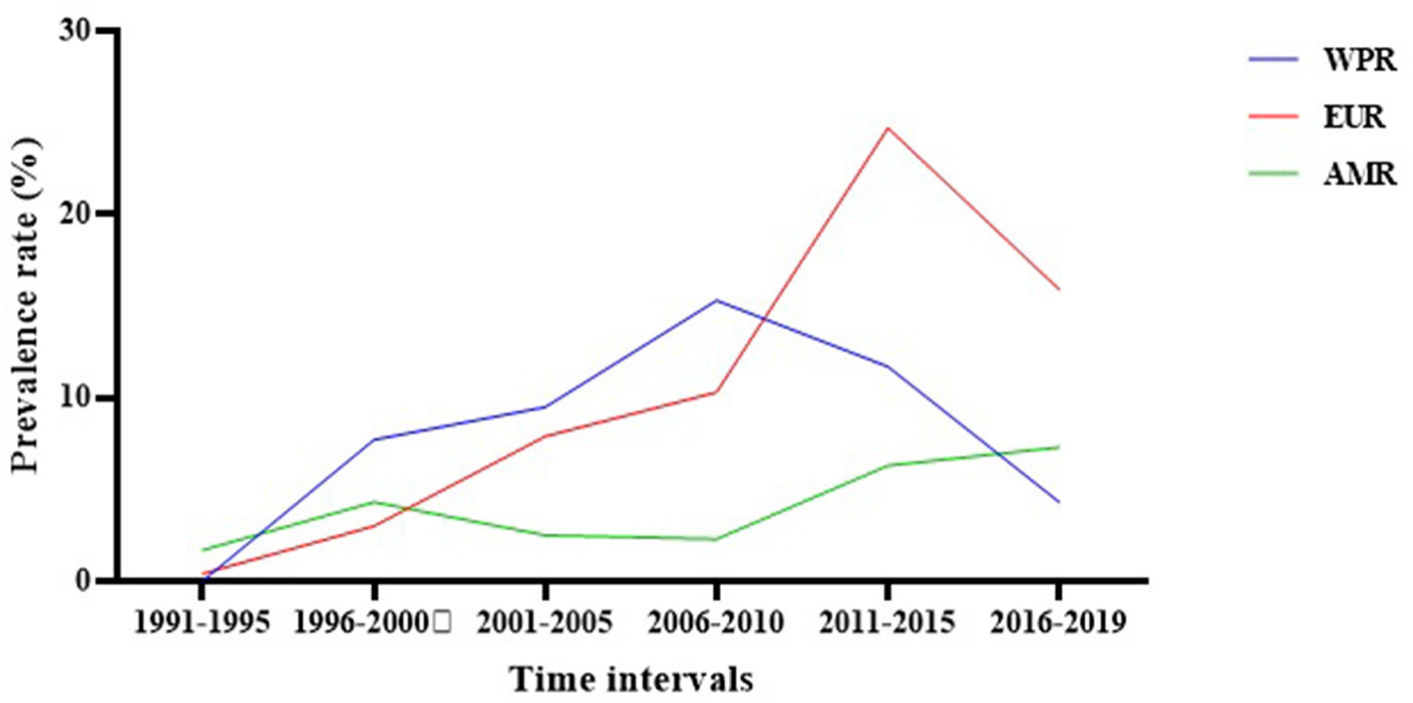
The regional prevalence of *S. maltophilia* isolation based on the publication time of studies.

Evaluation of the regional prevalence of *S. maltophilia* isolation based on the publication time of studies (from 1991 to 2019) showed an overall increasing trend. In the Western Pacific Region, the prevalence rate of *S. maltophilia* decreased from 2006 to 2010; however, the prevalence rates in the European Region and the Regions of America increased after this time interval (Figure 5 and Supplementary File 1).

### The antibiotic resistance rate of *Stenotrophomonas maltophilia*

The susceptibility of *S. maltophilia* isolates to various antibiotics was determined using various methods, including broth micro-dilution, broth macro-dilution, agar dilution, disk agar diffusion (DAD), E-test, and automated methods (e.g., VITEK, Phoenix, and micro-scan systems). Broth micro-dilution was the most frequently used assay. The standards used for interpreting the results of susceptibility assays varied, with different breakpoints used, such as those of the Clinical and Laboratory Standards Institute (CLSI), National Committee for Clinical Laboratory Standards (NCCLS), European Committee on Antimicrobial Susceptibility Testing (EUCAST), U.S. Food and Drug Administration (FDA), British Society for Antimicrobial Chemotherapy (BSAC), TRUST, and Comité de l'Antibiogramme de la Société Française de Microbiologie (CA-SFM) (Supplementary File 2).

As shown in Table 4, the highest resistance rates of *S. maltophilia* isolates were to cefuroxime [99.1%; 95% CI, 97.3–99.7%], cefoxitin [96.5%; 95% CI, 80.9–99.4%], ampicillin [96.1%; 95% CI, 92.8–97.9%], imipenem [94.9%; 95% CI, 92.3–96.7%], and meropenem [93.3%; 95% CI, 87.2–96.6%], while the lowest resistance rates were to doxycycline [5.7%; 95% CI, 3.3–9.7%] and minocycline [4.8%; 95% CI, 2.6–8.8%].

**Table 4:** Total antibiotic resistance rates of *Stenotrophomonas maltophilia* strains in the world.

**Antibiotic**	**No. of studies**	**Antibiotic resistance rate [95% CI]**	**N/total**	**Heterogeneity test, *I^2^***	**Heterogeneity test, *P-*value**	**Begg's test**	**Egger's test**
**Penicillins**							
Ampicillin	6	96.1 [92.8–97.9]	358/367	41.721	0.127	1.000	0.509
Ticarcillin	14	67.6 [53.5–79.1]	1126/1616	93.177	0.000	1.000	0.982
Piperacillin	29	72.5 [64.1–79.5]	2167/3108	93.636	0.000	0.652	0.251
**Cephalosporins**							
Ceftazidime	120	53.7 [49.8–57.5]	8445/17526	94.850	0.000	0.561	0.005
Cefoprazone	6	53 [29.6–75.2]	248/747	96.172	0.000	0.707	0.141
Cefepime	39	59.5 [50.7–67.8]	2310/4120	95.313	0.000	0.260	0.414
Cefoxitin	8	96.5 [80.9–99.4]	263/276	84.133	0.000	0.107	0.010
Cefotaxime	19	89.5 [77.8–95.4]	1093/1546	95.747	0.000	0.401	0.018
Ceftriaxone	24	91.2 [83.3–95.5]	1253/1588	91.399	0.000	0.172	0.051
Cefuroxime	6	99.1 [97.3–99.7]	528/529	0.000	0.796	0.132	0.663
β**-lactam/**β**-lactamase inhibitor**							
Amoxicillin/clavulanate	10	91 [73.5–97.4]	562/621	90.444	0.000	0.858	0.141
Ampicillin/sulbactam	4	91.7 [15.2–99.9]	128/372	93.917	0.000	1.000	0.004
Ticarcillin/clavulanate	54	33.2 [27.7–39.2]	3406/12314	96.699	0.000	0.665	0.137
Cefoprazone/sulbactam	7	30.7 [16.7–49.5]	165/936	92.308	0.000	0.229	0.040
Piperacillin/tazobactam	49	62.9 [55.6–69.6]	3135/5195	94.150	0.000	0.869	0.568
**Carbapenems**							
Meropenem	39	93.3 [87.2–96.6]	2574/3149	95.578	0.000	0.004	0.00024
Imipenem	64	94.9 [92.3–96.7]	4399/5203	92.250	0.000	0.013	0.000
**Monobactams**							
Aztreonam	24	84.1 [68.8–92.7]	1457/2662	97.164	0.000	0.711	0.038
**Aminoglycosides**							
Amikacin	59	69.8 [63.2–75.7]	3874/5783	94.439	0.000	0.432	0.483
Gentamicin	53	73.4 [66.4–79.3]	3077/4256	92.875	0.000	0.240	0.993
Tobramycin	26	81 [74.5–86.2]	1921/2483	88.506	0.000	0.122	0.179
Netilmicin	8	73.2 [46.2–89.7]	353/490	94.806	0.000	0.265	0.443
**Fluoroquinolones**							
Ciprofloxacin	100	47.6 [42.6–52.5]	4888/9660	93.837	0.000	0.114	0.628
Levofloxacin	72	19.7 [16.4–23.4]	2250/14141	94.656	0.000	0.046	0.607
Moxifloxacin	12	17.5 [9.8–29.2]	218/1858	93.896	0.000	0.890	0.224
Ofloxacin	16	29.9 [22.1–39]	546/1697	89.733	0.000	0.558	0.241
Gatifloxacin	7	10.9 [5.9–19.4]	220/2809	94.490	0.000	1.000	0.487
Norfloxacin	9	66.9 [45.3–83.1]	324/458	90.688	0.000	0.465	0.349
Trovafloxacin	6	16.3 [5.9–37.7]	153/1190	95.506	0.000	0.707	0.748
**Tetracyclines**							
Tetracycline	13	58.6 [45.2–70.8]	1398/2432	95.208	0.000	0.450	0.987
Doxycycline	10	5.7 [3.3–9.7]	189/2312	88.180	0.000	0.283	0.112
Minocycline	18	4.8 [2.6–8.8]	172/3018	91.488	0.000	0.288	0.00040
Tigecycline	18	11.8 [7–19.1]	474/3849	95.745	0.000	0.404	0.317
Chloramphenicol	29	46.9 [37.2–56.9]	2507/5223	97.284	0.000	0.735	0.719
**Polymyxins**							
Colistin	19	48.4 [31.6–65.5]	911/1768	95.839	0.000	1.000	0.213
High-dose colistin	5	27.3 [10.8–53.7]	488/1826	96.376	0.000	0.806	0.386
Polymyxin B	8	18 [11.8–26.5]	819/3896	94.518	0.000	1.000	0.411
**Sulfonamides**							
Trimethoprim/ sulfamethoxazole	93	14.7 [11.7–18.3]	2968/20084	96.824	0.000	0.611	0.010
**Phosphonic antibiotics**							
Fosfomycin	6	32.3 [12.4–61.7]	223/818	97.308	0.000	1.000	0.759

**Figure 6:**
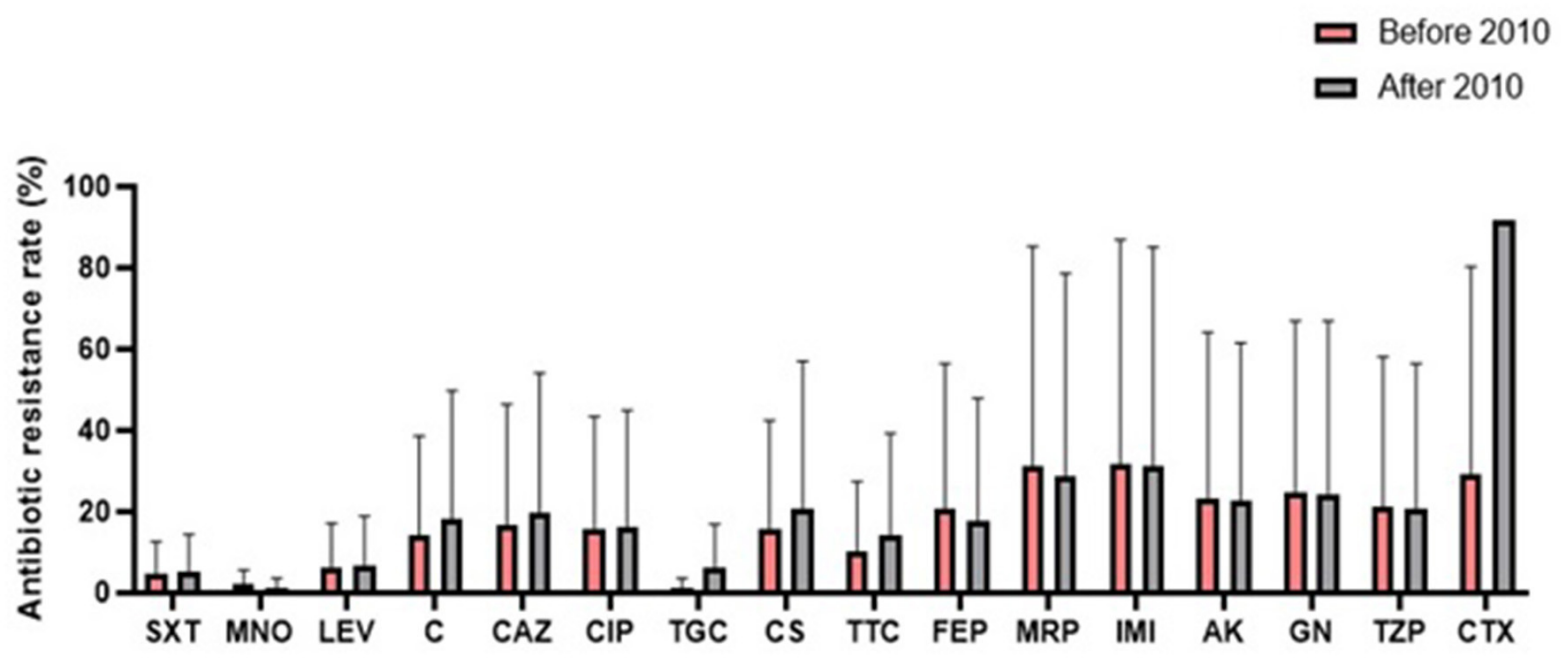
Comparison of the global antibiotic resistance rates of *S. maltophilia* before and after 2010 (SXT, trimethoprim-sulfamethoxazole; MNO, minocycline; LEV, levofloxacin; C, chloramphenicol; CAZ, ceftazidime; CIP, ciprofloxacin; TGC, tigecycline; CS, colistin; TTC, ticarcillin-clavulanic acid; FEP, cefepime; MRP, meropenem; IMI, imipenem; AK, amikacin; GN, gentamicin; TZP, piperacillin-tazobactam; CTX, cefotaxime).

A comparison of antibiotic resistance rates of *S. maltophilia* before and after 2010 (Figure 6) revealed an increasing trend for some antibiotics, such as chloramphenicol (12.3%), TMP/SMX (11.6%), ceftazidime (8.6%), and levofloxacin (1.8%). Conversely, the resistance rate against minocycline (2.2%) decreased.

**Figure 7:**
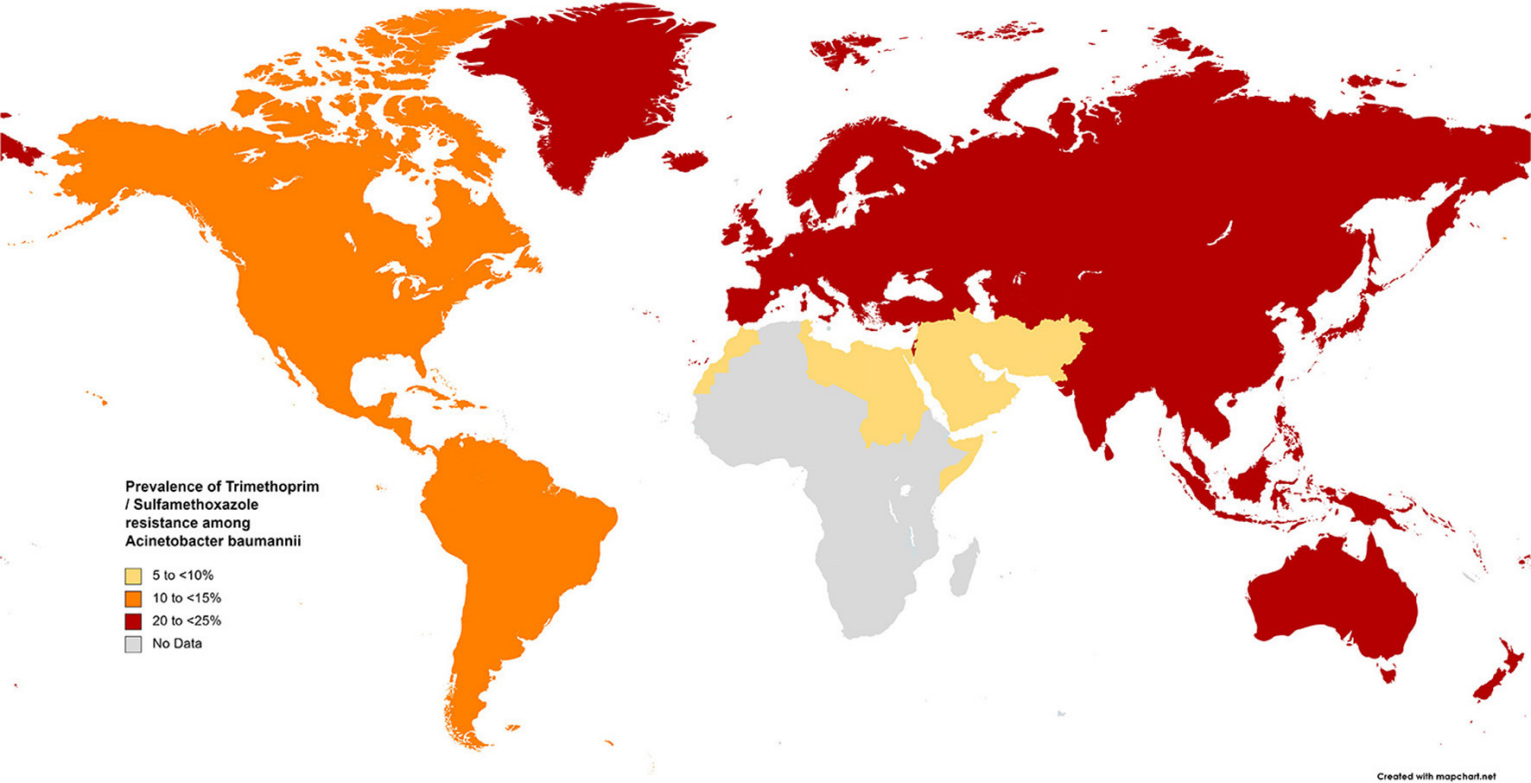
Prevalence of trimethoprim/sulfamethoxazole resistance in *S. maltophilia* isolated from clinical samples, by WHO regions.

**Figure 8:**
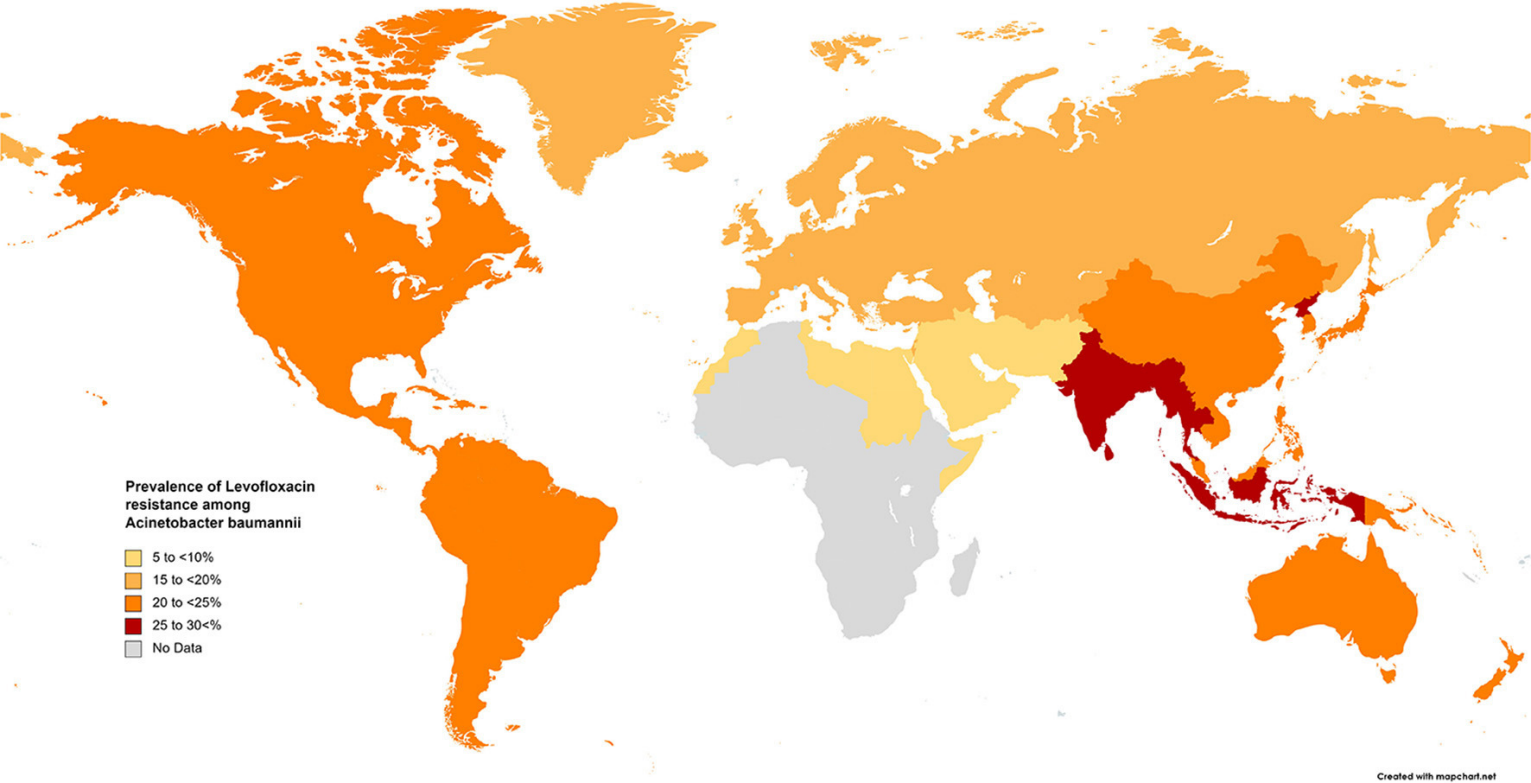
Prevalence of levofloxacin resistance in *S. maltophilia* isolated from clinical samples, by WHO regions.

**Figure 9:**
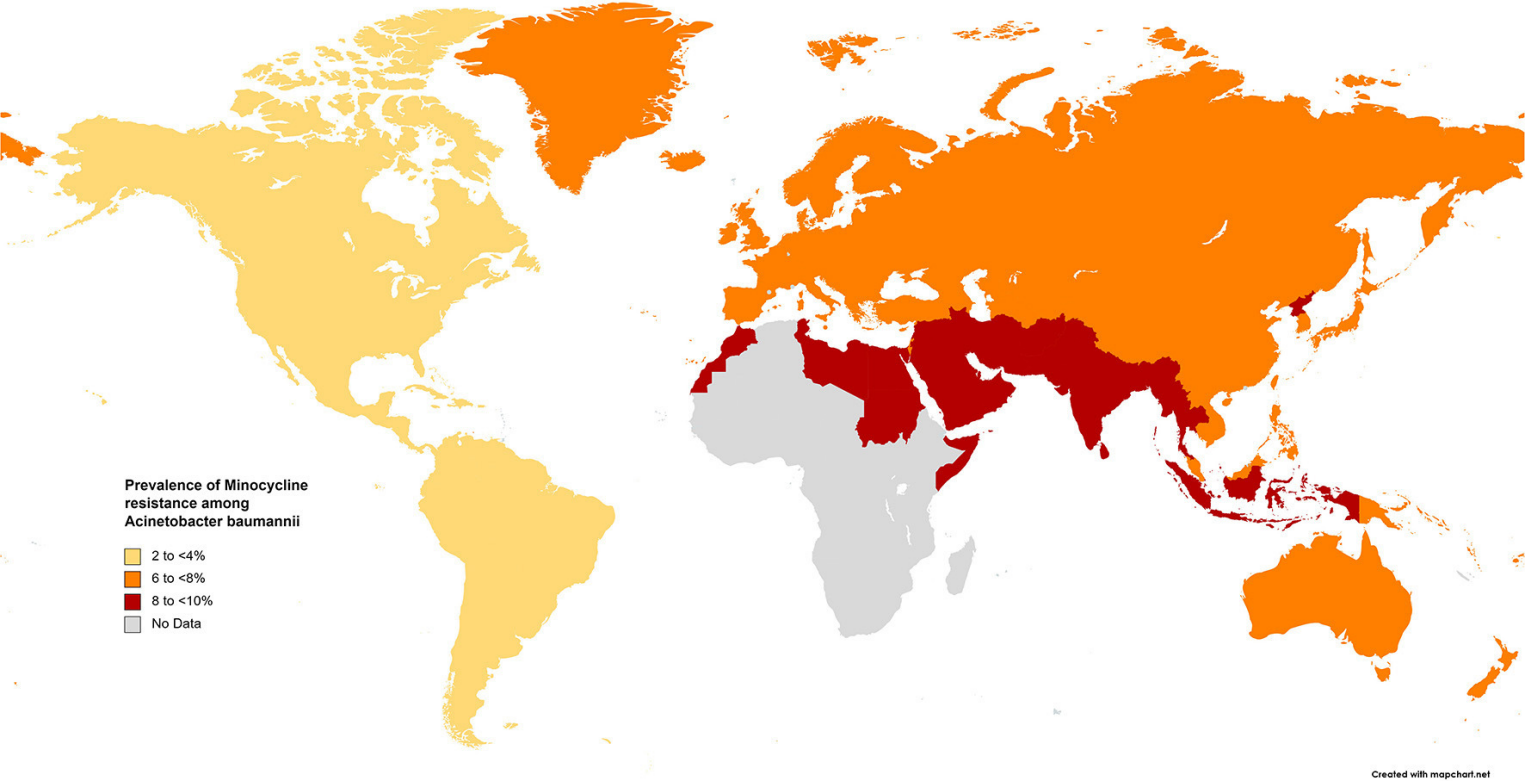
Prevalence of minocycline resistance in *S. maltophilia* isolated from clinical samples, by WHO regions.

The results of the subgroup meta-analysis based on the world regions and antibiotic resistance rates, presented in Figures 7–9, as well as in Supplementary File 1, showed that the highest resistance rate across all regions was to ceftazidime, while the lowest rate was to minocycline.

## Discussion

Although *S. maltophilia* shows limited invasiveness in immunocompetent individuals, it can lead to severe infections in immunocompromised patients. Moreover, its high intrinsic resistance to a large number of antimicrobial agents results in treatment failure and mortality in patients infected by this microorganism (191–194). Thus, the undertaking of a first systematic review and meta-analysis addressing the prevalence rate of isolation and antibiotic resistance rates of *S. maltophilia* in different regions of the world may be of great value in managing infections caused by this bacterium.

Based on the present meta-analysis, most studies were reported from the European Region (*n* = 57, 32%), while in a similar investigation (12), the majority of cases were reported and managed in the United States of America (*n* = 72, 27.7%). The differences between the inclusion and exclusion criteria applied in these two studies may explain the differing results. In the current study, the global prevalence rate of *S. maltophilia* isolation from clinical samples was 5.3%, and according to the WHO classification, the highest prevalence rate of *S. maltophilia* isolation was observed in the Western Pacific Region (10.5%), followed by the European Region (7.9%), which may be due to their long-shared land border. Among the reasons for the discrepancies in the prevalence of *Stenotrophomonas maltophilia* infection in different world regions, we can mention the following: disparate health policies in each country affect the importance of pathogens, so, in some countries, *Stenotrophomonas maltophilia* is still considered an unimportant opportunistic pathogen, so few studies have been reported. For example, most of the cases were documented in European (195), Asian (86), and American (196) countries, while there was no relevant study performed in the African continent. This difference can cause publication bias and affect the overall results. Additionally, the differences in health levels of various countries and the numbers and types of examined patients all influence the reported prevalence of *Stenotrophomonas maltophilia*.

In this meta-analysis, among different clinical samples, respiratory samples were the most frequent source (67%), followed by blood samples (24%). This finding is consistent with other studies, in which *S. maltophilia* was most commonly associated with respiratory tract infections, followed by bloodstream infections (74, 197). However, in another systematic review, blood was the most prevalent site of *S. maltophilia* isolation (12). In a large study performed in the USA and fifteen centers in European countries in 2012, 6.3% of the isolates obtained from respiratory tract infections were identified as *S. maltophilia*. These data suggest that the rate of respiratory tract infections caused by *S. maltophilia* is increasing (3, 198). The bacterium's capability for adherence to plastic surfaces and biofilm formation on hospital devices, such as those inserted into the respiratory tract, may explain its high rate in the aforementioned samples (199, 200). For example, among patients with ventilator-associated pneumonia (VAP), the most common nosocomial infection in mechanically ventilated patients, *S. maltophilia* is the probable causative pathogen (196, 201). Moreover, its adaptation to the airways of individuals with cystic fibrosis (CF) has led it to being recognized as an emerging multi-drug resistant opportunistic pathogen (86).

The prevalence rate of infections caused by this bacterium increased from 1.7% to 6.5% during the 31 investigated years, suggesting that it is emerging as an opportunistic pathogen, particularly among immunocompromised hosts. This rapid rise may be due to its resistance to a wide range of antimicrobial agents, as well as the increased focus on this bacterium as a cause of infection. The treatment of *S. maltophilia* infections is challenging due to the difficulty of differentiating colonization from infection and the intrinsic resistance of this bacterium to multiple classes of antibiotics. The WHO has classified *S. maltophilia* as one of the leading multidrug-resistant organisms in hospital settings (202). Additionally, recent antibiotic treatment and other known factors associated with acquiring *S. maltophilia* infections demonstrate specific features of this bacterium (195).

Based on our data, the highest and the lowest global resistant rates were to cefuroxime and minocycline, respectively (Figure 3). The lowest resistance to TMP-SMX was observed in the EMR (4.5%) and AMR (13.1%), while in other geographical regions, resistance was higher than 20%. Consequently, TMP-SMX may be the first choice for treatment based on antibiotic susceptibility and therapeutic success (3, 60, 203). Fortunately, in the present study, a comparison of global antibiotic resistance rates of *S. maltophilia* before and after 2010 (Figure 4) confirmed the effectiveness of this medication for treating infections of this opportunistic organism. However, there is not always a logical correlation between laboratory sensitivity and clinical results. Other antibiotics for treating *Stenotrophomonas* infections include fluoroquinolones, tetracyclines, and selected β-lactams, such as ceftazidime and ticarcillin/clavulanate. However, the development of resistance to some of these antibiotics renders them unreliable.

Fluoroquinolones are prescribed for treating infections caused by TMP-SMX-resistant *S. maltophilia* and for patients for whom this drug has adverse effects. Studies comparing treatments with fluoroquinolones and TMP-SMX have proposed that levofloxacin has similar effectiveness with fewer adverse effects than TMP-SMX (204, 205). Our study indicates that resistance rates to levofloxacin vary geographically, ranging from 6.4% in EMR to 15%−22% in EUR, AMR, and WPR, and up to 26% in SEAR. However, the rapid emergence of resistance against quinolones *in vitro* and *in vivo* is of concern when levofloxacin is used to treat *S. maltophilia* infections.

In surveillance studies of the efficacy of tigecycline and related tetracycline antibiotics, minocycline was found to be effective against *S. maltophilia* (206). In this study, resistance to minocycline was <10% in all geographical areas and global resistance to tigecycline was 11.8%. A comparison of the antibiotic resistance rates of *S. maltophilia* before and after 2010 revealed an increase in resistance to tigecycline from 4.1% to 18.6%. Several studies have revealed that minocycline is not inferior to TMP-SMX and may even be more suitable than TMP-SMX in terms of susceptibility. These results suggest that minocycline and TMP-SMX may be the first-line therapy in *S. maltophilia* infections, even in TMP-SMX-resistant strains (59).

Ceftazidime and ticarcillin/clavulanate have previously been reported as the most effective β-lactam drugs against *S. maltophilia*. However, reduced sensitivity to ceftazidime has been documented in recent studies. Owing to β-lactamase production, a high resistance rate to β-lactams such as cefuroxime, cefoxitin, imipenem, and meropenem (> 90%, Table 4) has been observed, thus reducing their role in the treatment of *S. maltophilia* infections (207). According to this analysis, ceftazidime has a high resistance rate in all regions classified by the WHO (AMR, 56.4%; EMR, 42.9%; SEAR, 65.1%; WPR, 52.6%). Our study suggests that the rate of resistance to ticarcillin/clavulanate globally is 33.2%. Therefore, these current resistance rates to ceftazidime and ticarcillin/clavulanate render them unreliable. However, the use of ceftazidime in combination with other antibiotics (typically vancomycin, amikacin, TMP-SMX, or fluoroquinolones) is an effective treatment for infections caused by *S. maltophilia* (13). A systemic literature review by Gibb and Wong (208) offers recommendations for a treatment strategy for *Stenotrophomonas* infection based on current evidence. The first-line drugs suggested are TMP-SMX, fluoroquinolones, and tetracyclines.

Our study presents several limitations. First, a large number of the included studies (84 articles) evaluated a specific number of *S. maltophilia* isolates but did not report the prevalence rate of isolation; thus, these studies were not included in the meta-analysis, which could affect the pooled prevalence rate of *S. maltophilia* isolation and the antibiotic resistance rates. Second, the number of published studies reporting the resistance mechanism of strains isolated from clinical samples (see Supplementary File 2) is relatively small, and the specific genes conferring antibiotic resistance in these isolates remain unclear. Third, a few studies used typing methods to evaluate *S. maltophilia* isolates (see Supplementary File 2), so we could not report the most prevalent types of this bacterium at the global and regional levels.

## Conclusion

In conclusion, despite the undeniable clinical impact of *S. maltophilia*, compared with other Gram-negative species, this bacterium is remarkably understudied. Thus, collecting and analyzing data related to different aspects of *S. maltophilia* may assist in improving the clinical management of challenges caused by this bacterium. This meta-analysis presents the global antibiotic resistance of *S. maltophilia* over the last 31 years and demonstrates different rates of resistance in world geographical regions, as well as the growing trend of resistance to most antibiotics. The variations in antibiotic resistance of *S. maltophilia* isolates in different regions may be the result of the use of different protocols for patient treatment. Additionally, the improper and experimental use of antibiotics plays an important role in increasing resistance, leading to an increased risk of treatment failure. To address this issue, it is necessary to carry out antibiotic sensitivity tests before prescribing antibiotics and implementing an antimicrobial stewardship program for every hospital, as well as provide continuous training for clinicians about their performance in the hospital environment. Finally, collecting and preparing local sensitivity patterns will be effective in allowing the selection of the optimal empiric treatment for *S. maltophilia* infections.

## Author contributions

MB contributed to the study design, data extraction, data analysis, design and production of figures, and wrote and revised the final manuscript. AS-M contributed to the study design, data extraction, data analysis, and writing of the manuscript. GB contributed to the data analysis and statistical analysis, designed and produced figures, and writing of the manuscript. EE contributed to the study design, data extraction, and writing of the manuscript. LJ contributed to the study design and the writing and revision of the final manuscript. RB contributed to the study design, data analysis and interpretation, and the writing of the manuscript. ME designed the study, oversaw the analysis, and wrote and revised the final manuscript. FJ designed the study, was the arbiter for the study searches and data extraction, and wrote and revised the final manuscript. All authors contributed to the article and approved the submitted version.
