# Supplementary material for: Global prevalence and antibiotic resistance in clinical isolates of Stenotrophomonas maltophilia: a systematic review and meta-analysis
Source: Front Med (Lausanne). 2023 May 5;10:1163439. doi: 10.3389/fmed.2023.1163439 (PMC10196134; doi:10.3389/fmed.2023.1163439)
Supplement: Supplementary file 1 [file Data_Sheet_1.docx]

**List of Contents**

[**Funnel plot of the meta-analysis for the global prevalence rate of *S. maltophilia* isolation from clinical samples** 2](#_Toc132722876)

[**Details of the subgroup meta-analysis, the forest plots, and the funnel plots of the global prevalence of *S. maltophilia* isolation in five years’ time intervals** 3](#_Toc132722877)

[**Details of the subgroup meta-analysis, the forest plots, and the funnel plots of the prevalence of *S. maltophilia* isolation in five years’ time intervals in the West Pacific Region** 15](#_Toc132722889)

[**Details of the subgroup meta-analysis, the forest plots, and the funnel plots of the prevalence of *S.* *maltophilia* isolation in five years’ time intervals in the European Region** 22](#_Toc132722896)

[**Details of the subgroup meta-analysis, the forest plots, and the funnel plots of the prevalence of *S. maltophilia* isolation in five years’ time intervals in Regions of America** 33](#_Toc132722907)

[**Details of meta-analysis for antibiotic resistance rates of *S. maltophilia* in different world regions** 44](#_Toc132722918)

# **Funnel plot of the meta-analysis for the global prevalence rate of *S. maltophilia* isolation from clinical samples**

# **Details of the subgroup meta-analysis, the forest plots, and the funnel plots of the global prevalence of *S. maltophilia* isolation in five years’ time intervals**

| Table1. Meta-analysis of the global prevalence of *Stenotrophomonas maltophilia* from 1991-1995 | | | | | | | |
| --- | --- | --- | --- | --- | --- | --- | --- |
| Subgroups | No. of studies | Prevalence of *S. maltophilia* | n/N | Heterogeneity test, *I^2^* | Heterogeneity test, *P* value | Begg’s test | Egger’s test |
| Overall | 13 | 1.7 (0.7-4) | 696/157899 | 99.155 | 0.000 | 0.502 | 0.036 |

| Table 2. Meta-analysis of the global prevalence of *Stenotrophomonas maltophilia* from 1996-2000 | | | | | | | |
| --- | --- | --- | --- | --- | --- | --- | --- |
| Subgroups | No. of studies | Prevalence of *S.* *maltophilia* | n/N | Heterogeneity test, *I^2^* | Heterogeneity test, *P* value | Begg’s test | Egger’s test |
| Overall | 11 | 4.5 (2.2-8.8) | 569/38696 | 98.493 | 0.000 | 0.119 | 0.003 |

| Table 3. Meta-analysis of the global prevalence of *Stenotrophomonas maltophilia* from 2001-2005 | | | | | | | |
| --- | --- | --- | --- | --- | --- | --- | --- |
| Subgroups | No. of studies | Prevalence of *S.* *maltophilia* | n/N | Heterogeneity test, *I^2^* | Heterogeneity test, *P* value | Begg’s test | Egger’s test |
| Overall | 17 | 4.4 (2.7-7.1) | 4159/156226 | 99.525 | 0.000 | 0.232 | 0.983 |


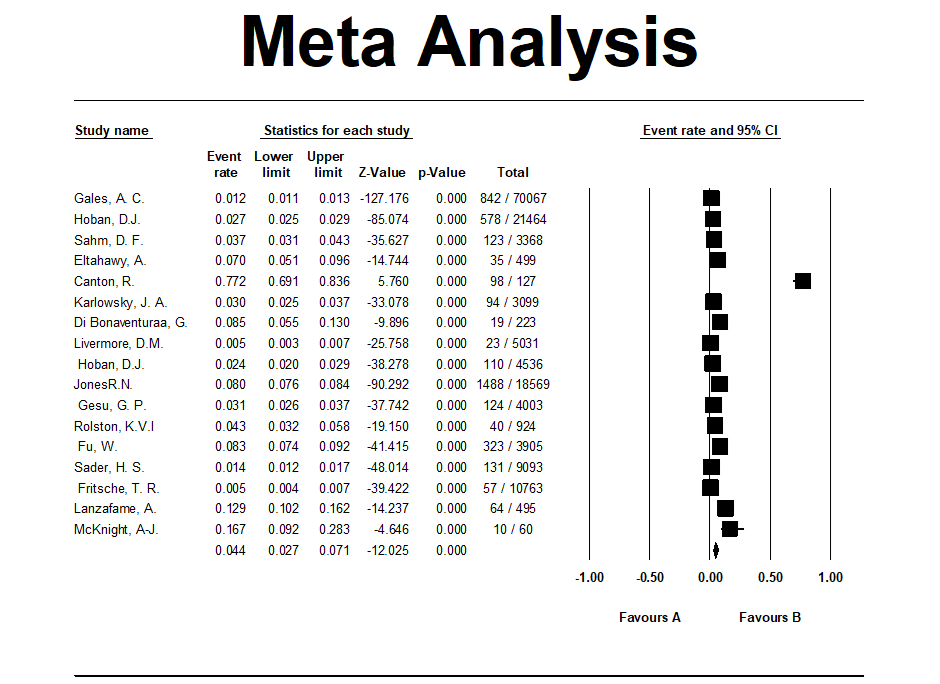

| Table 4. Meta-analysis of the global prevalence of *Stenotrophomonas maltophilia* from 2006-2010 | | | | | | | |
| --- | --- | --- | --- | --- | --- | --- | --- |
| Subgroups | No. of studies | Prevalence of *S.* *maltophilia* | n/N | Heterogeneity test, *I^2^* | Heterogeneity test, *P* value | Begg’s test | Egger’s test |
| Overall | 13 | 7.4 (4.5-12.1) | 1834/28534 | 98.529 | 0.000 | 0.951 | 0.620 |

| Table 5. Meta-analysis of the global prevalence of *Stenotrophomonas maltophilia* from 2011-2015 | | | | | | | |
| --- | --- | --- | --- | --- | --- | --- | --- |
| Subgroups | No. of studied | Prevalence of *S.* *maltophilia* | n/N | Heterogeneity test, *I^2^* | Heterogeneity test, *P* value | Begg’s test | Egger’s test |
| Overall | 20 | 7.7 (4.3-13.4) | 2465/135819 | 99.517 | 0.000 | 0.047 | 0.011 |

| Table 6. Meta-analysis of the global prevalence of *Stenotrophomonas maltophilia* from 2016-2019 | | | | | | | |
| --- | --- | --- | --- | --- | --- | --- | --- |
| Subgroups | No. of studies | Prevalence of *S.* *maltophilia* | n/N | Heterogeneity test, *I^2^* | Heterogeneity test, *P* value | Begg’s test | Egger’s test |
| Overall | 20 | 6.5 (4.1-10.1) | 1383/43283 | 98.555 | 0.000 | 0.381 | 0.157 |

# **Details of the subgroup meta-analysis, the forest plots, and the funnel plots of the prevalence of *S. maltophilia* isolation in five years’ time intervals in the West Pacific Region**

| Table1. Meta-analysis of prevalence of *Stenotrophomonas maltophilia* in WPR from 1996-2000 | | | | | | | |
| --- | --- | --- | --- | --- | --- | --- | --- |
| Subgroups | No. of studies | Prevalence of *S.* *maltophilia* | n/N | Heterogeneity test, *I^2^* | Heterogeneity test, *P* value | Begg’s test | Egger’s test |
| Overall | 1 | 7.7 (5.3-10.9) | 28/366 | 0.000 | 1.000 | - | - |

| Table2. Meta-analysis of prevalence of *Stenotrophomonas maltophilia* in WPR from 2001-2005 | | | | | | | |
| --- | --- | --- | --- | --- | --- | --- | --- |
| Subgroups | No. of studies | Prevalence of *S.* *maltophilia* | n/N | Heterogeneity test, *I^2^* | Heterogeneity test, *P* value | Begg’s test | Egger’s test |
| Overall | 2 | 9.5 (6.9-12.8) | 373/4345 | 79.053 | 0.029 | - | - |

| Table3. Meta-analysis of prevalence of *Stenotrophomonas maltophilia* in WPR from 2006-2010 | | | | | | | |
| --- | --- | --- | --- | --- | --- | --- | --- |
| Subgroups | No. of studies | Prevalence of *S.* *maltophilia* | n/N | Heterogeneity test, *I^2^* | Heterogeneity test, *P* value | Begg’s test | Egger’s test |
| Overall | 2 | 15.3 (12.2-19.1) | 65/425 | 0.000 | 0.659 | - | - |

| Table 4. Meta-analysis of prevalence of *Stenotrophomonas maltophilia* in WPR from 2011-2015 | | | | | | | |
| --- | --- | --- | --- | --- | --- | --- | --- |
| Subgroups | No. of studies | Prevalence of *S.* *maltophilia* | n/N | Heterogeneity test, *I^2^* | Heterogeneity test, *P* value | Begg’s test | Egger’s test |
| Overall | 4 | 11.7 (3.6-31.5) | 126/1762 | 97.059 | 0.000 | 1.000 | 0.464 |

| Table 5. Meta-analysis of prevalence of *Stenotrophomonas maltophilia* in WPR from 2016-2019 | | | | | | | |
| --- | --- | --- | --- | --- | --- | --- | --- |
| Subgroups | No. of studies | Prevalence of *S.* *maltophilia* | n/N | Heterogeneity test*, I^2^* | Heterogeneity test, *P* value | Begg’s test | Egger’s test |
| Overall | 3 | 4.3 (1.3-13.8) | 100/2194 | 97.029 | 0.000 | 0.296 | 0.326 |

| **Details of the subgroup meta-analysis, the forest plots, and the funnel plots of the prevalence of *S.* *maltophilia* isolation in five years’ time intervals in the European Region** Table1. Meta-analysis of prevalence of *Stenotrophomonas maltophilia* in Europe from 1991-1995 | | | | | | | |
| --- | --- | --- | --- | --- | --- | --- | --- |
| Subgroups | No. of studies | Prevalence of *S.* *maltophilia* | n/N | Heterogeneity test, *I^2^* | Heterogeneity test, *P* value | Begg’s test | Egger’s test |
| Overall | 3 | 0.4 (0.1-0.9) | 276/139183 | 96.082 | 0.000 | 0.296 | 0.305 |

| Table 2. Meta-analysis of prevalence of *Stenotrophomonas maltophilia* in Europe from 1996-2000 | | | | | | | |
| --- | --- | --- | --- | --- | --- | --- | --- |
| Subgroups | No. of studies | Prevalence of *S.* *maltophilia* | n/N | Heterogeneity test, *I^2^* | Heterogeneity test, *P* value | Begg’s test | Egger’s test |
| Overall | 2 | 3 (0.4-19.6) | 131/9985 | 98.789 | 0.000 | - | - |

| Table 3. Meta-analysis of prevalence of *Stenotrophomonas maltophilia* in Europe from 2001-2005 | | | | | | | |
| --- | --- | --- | --- | --- | --- | --- | --- |
| Subgroups | No. of studies | Prevalence of *S.* *maltophilia* | n/N | Heterogeneity test, *I^2^* | Heterogeneity test, *P* value | Begg’s test | Egger’s test |
| Overall | 7 | 7.9 (3-19.4) | 916/31403 | 99.201 | 0.000 | 0.229 | 0.235 |

| Table 4. Meta-analysis of prevalence of *Stenotrophomonas maltophilia* in Europe from 2006-2010 | | | | | | | |
| --- | --- | --- | --- | --- | --- | --- | --- |
| Subgroups | No. of studies | Prevalence of *S.* *maltophilia* | n/N | Heterogeneity test, *I^2^* | Heterogeneity test, *P* value | Begg’s test | Egger’s test |
| Overall | 4 | 10.3 (3.4-27.2) | 207/2064 | 98.240 | 0.000 | 0.734 | 0.242 |

| Table 5. Meta-analysis of prevalence of *Stenotrophomonas maltophilia* in Europe from 2011-2015 | | | | | | | |
| --- | --- | --- | --- | --- | --- | --- | --- |
| Subgroups | No. of studies | Prevalence of *S.* *maltophilia* | n/N | Heterogeneity test, *I^2^* | Heterogeneity test, *P* value | Begg’s test | Egger’s test |
| Overall | 6 | 24.7 (8-55.2) | 462/5186 | 99.179 | 0.000 | 0.060 | 0.041 |

| Table 6. Meta-analysis of prevalence of *Stenotrophomonas maltophilia* in Europe from 2016-2019 | | | | | | | |
| --- | --- | --- | --- | --- | --- | --- | --- |
| Subgroups | No. of studies | Prevalence of *S*. *maltophilia* | n/N | Heterogeneity test, *I^2^* | Heterogeneity test, *P* value | Begg’s test | Egger’s test |
| Overall | 4 | 15.9 (5.8-36.8) | 124/1221 | 96.812 | 0.000 | 0.734 | 0.573 |

# **Details of the subgroup meta-analysis, the forest plots, and the funnel plots of the prevalence of *S. maltophilia* isolation in five years’ time intervals in Regions of America**

| Table1. Meta-analysis of prevalence of *Stenotrophomonas maltophilia* in America from1991-1995 | | | | | | | |
| --- | --- | --- | --- | --- | --- | --- | --- |
| Subgroups | No. of studies | Prevalence of *S.* *maltophilia* | n/N | Heterogeneity test, *I^2^* | Heterogeneity test, *P* value | Begg’s test | Egger’s test |
| Overall | 2 | 1.7 (0.9-3.2) | 86/4269 | 72.981 | 0.000 | - | - |

| Table 2. Meta-analysis of prevalence of *Stenotrophomonas maltophilia* in America from1996-2000 | | | | | | | |
| --- | --- | --- | --- | --- | --- | --- | --- |
| Subgroups | No. of studies | Prevalence of *S.* *maltophilia* | n/N | Heterogeneity test, *I^2^* | Heterogeneity test, *P* value | Begg’s test | Egger’s test |
| Overall | 6 | 4.3 (1.7-10.8) | 399/28932 | 98.715 | 0.000 | 0.132 | 0.095 |

| Table 3. Meta-analysis of prevalence of *Stenotrophomonas maltophilia* in America from 2001-2005 | | | | | | | |
| --- | --- | --- | --- | --- | --- | --- | --- |
| Subgroups | No. of studies | Prevalence of *S*. *maltophilia* | n/N | Heterogeneity test, *I^2^* | Heterogeneity test, *P* value | Begg’s test | Egger’s test |
| Overall | 4 | 2.5 (1.5-4.2) | 489/27097 | 96.578 | 0.000 | 0.089 | 0.046 |

| Table 4. Meta-analysis of prevalence of *Stenotrophomonas maltophilia* in America from 2006-2010 | | | | | | | |
| --- | --- | --- | --- | --- | --- | --- | --- |
| Subgroups | No. of studies | Prevalence of *S.* *maltophilia* | n/N | Heterogeneity test, *I^2^* | Heterogeneity test, *P* value | Begg’s test | Egger’s test |
| Overall | 3 | 2.3 (1.8-3) | 305/13114 | 81.505 | 0.000 | 1.000 | 0.258 |

| Table 5. Meta-analysis of prevalence of *Stenotrophomonas maltophilia* in America from 2011-2015 | | | | | | | |
| --- | --- | --- | --- | --- | --- | --- | --- |
| Subgroups | No. of studies | Prevalence of *S*. *maltophilia* | n/N | Heterogeneity test, *I^2^* | Heterogeneity test, *P* value | Begg’s test | Egger’s test |
| Overall | 4 | 6.3 (1.8-20.2) | 321/12445 | 99.094 | 0.000 | 0.089 | 0.131 |

| Table 6. Meta-analysis of prevalence of *Stenotrophomonas maltophilia* in world from 2016-2019 | | | | | | | |
| --- | --- | --- | --- | --- | --- | --- | --- |
| Subgroups | No. of studies | Prevalence of *S.* *maltophilia* | n/N | Heterogeneity test, *I^2^* | Heterogeneity test, *P* value | Begg’s test | Egger’s test |
| Overall | 7 | 7.3 (4.7-11.2) | 993/21459 | 96.719 | 0.000 | 0.133 | 0.095 |

# **Details of meta-analysis for antibiotic resistance rates of S. maltophilia in different world regions**

| Table1. Status of antibiotic-resistance among *Stenotrophomonas maltophilia* in Eastern Mediterranean Region | | | | | | | |
| --- | --- | --- | --- | --- | --- | --- | --- |
| Antibiotic | No. of studies | Antibiotic resistance rate (95% CI) | n/N | Heterogeneity test, *I^2^* | Heterogeneity test, *P* value | Begg’s test | Egger’s test |
| Trimethoprim/ sulfamethoxazole | 4 | 5.4 (3.9-7.5) | 33/609 | 0.000 | 0.989 | 0.734 | 0.618 |
| Minocycline | 1 | 8.5 (5.1-13.9) | 14/164 | 0.000 | 1.000 | - | - |
| Ceftazidime | 13 | 42.9 (26.4-61.1) | 341/870 | 94.219 | 0.000 | 0.582 | 0.598 |
| Ciprofloxacin | 10 | 16.1 (7.9-30) | 68/584 | 86.816 | 0.000 | 0.283 | 0.145 |
| Levofloxacin | 2 | 6.4 (0.1-86.3) | 11/190 | 94.534 | 0.000 | - | - |
| Colistin | 1 | 26.9 (13.4-46.7) | 7/26 | 0.000 | 1.000 | - | - |
| Aztreonam | 2 | 74.8 (19-97.4) | 36/44 | 87.859 | 0.004 | - | - |
| Cefepime | 1 | 45.5 (26.5-65.9) | 10/22 | 0.000 | 1.000 | - | - |
| Piperacillin/ Tazobactam | 3 | 14.8 (0.2-93.7) | 25/316 | 96.465 | 0.000 | 1.000 | 0.623 |
| Meropenem | 2 | 93.9 (82.3-98.1) | 181/190 | 63.891 | 0.096 | - | - |
| Imipenem | 10 | 95.6 (78.4-99.2) | 497/699 | 93.353 | 0.000 | 0.283 | 0.00002 |
| Amikacin | 10 | 52.9 (43.9-61.7) | 256/562 | 68.885 | 0.001 | 0.591 | 0.002 |
| Gentamicin | 10 | 51.4 (31-71.4) | 184/562 | 93.272 | 0.000 | 0.720 | 0.077 |
| Ticarcillin | 1 | 83.9 (66.6-93.1) | 26/31 | 0.000 | 1.000 | - | - |
| Piperacillin | 7 | 84.8 (72.3-92.2) | 197/228 | 69.511 | 0.003 | 0.367 | 0.906 |
| Ampicillin/ sulbactam | 1 | 13.2 (9.7-17.6) | 37/281 | 0.000 | 1.000 | - | - |
| Cefoxitin | 5 | 97.8 (94.2-99.2) | 182/184 | 0.000 | 0.982 | 0.806 | 0.027 |
| Cefotaxime | 5 | 88.8 (65.2-97.1) | 145/159 | 80.544 | 0.000 | 0.806 | 0.314 |
| Ceftriaxone | 3 | 64.7 (39.8-83.6) | 238/312 | 81.153 | 0.005 | 1.000 | 0.264 |
| Cefuroxime | 2 | 94.1 (75-98.8) | 35/36 | 14.943 | 0.278 | - | - |
| Tetracycline | 1 | 20.1 (14.7-27) | 33/164 | 0.000 | 1.000 | - | - |
| Amoxicillin/ clavulanate | 2 | 51.5 (24.8-77.3) | 20/36 | 56.009 | 0.132 | - | - |

| Table2. Status of antibiotic-resistance among  *Stenotrophomonas maltophilia* in South-East Asian Region | | | | | | | |
| --- | --- | --- | --- | --- | --- | --- | --- |
| Antibiotic | No. of studies | Antibiotic resistance rate (95% CI) | n/N | Heterogeneity test, *I^2^* | Heterogeneity test, *P* value | Begg’s test | Egger’s test |
| Trimethoprim/ sulfamethoxazole | 5 | 20.2 (15.2-26.5) | 40/210 | 32.538 | 0.204 | 0.806 | 0.120 |
| Minocycline | 1 | 8.5 (5.1-13.9) | 14/164 | 0.000 | 1.000 | - | - |
| Ceftazidime | 3 | 65.1 (33.8-87.2) | 113/182 | 91.637 | 0.000 | 1.000 | 0.894 |
| Ciprofloxacin | 3 | 33.2 (8.4-72.9) | 28/91 | 82.576 | 0.003 | 1.000 | 0.936 |
| Levofloxacin | 4 | 26.3 (8.8-56.9) | 41/195 | 90.923 | 0.000 | 0.308 | 0.385 |
| Chloramphenicol | 2 | 44.2 (36.9-51.8) | 75/170 | 44.878 | 0.178 | - | - |
| Colistin | 1 | 79.7 (68.1-87.8) | 51/64 | 0.000 | 1.000 | - | - |
| Cefoprazone/ Sulbactam | 1 | 33.3 (14.6-59.4) | 5/15 | 0.000 | 1.000 | - | - |
| Cefepime | 2 | 80.8 (51.2-94.4) | 41/49 | 67.367 | 0.080 | - | - |
| Piperacillin/ Tazobactam | 2 | 68.3 (57.1-77.8) | 52/76 | 0.000 | 0.595 | - | - |
| Meropenem | 1 | 83.3 (52.3-95.8) | 10/12 | 0.000 | 1.000 | - | - |
| Imipenem | 1 | 83.3 (52.3-95.8) | 10/12 | 0.000 | 1.000 | - | - |
| Doxycycline | 1 | 3.1 (0.8-11.7) | 2/64 | 0.000 | 1.000 | - | - |
| Ofloxacin | 1 | 75 (44.8-91.7) | 9/12 | 0.000 | 1.000 | - | - |
| Amikacin | 2 | 62.9 (51.5-73.1) | 48/76 | 0.000 | 0.360 | - | - |
| Gentamicin | 2 | 67 (55.7-76.6) | 51/76 | 0.000 | 0.528 | - | - |
| Ticarcillin | 1 | 29.2 (21.4-38.6) | 31/106 | 0.000 | 1.000 | - | - |
| Cefotaxime | 1 | 6.8 (4.4-10.4) | 19/281 | 0.000 | 1.000 | - | - |

| Table3. Status of antibiotic-resistance among *Stenotrophomonas maltophilia* in Western Pacific Region. | | | | | | | |
| --- | --- | --- | --- | --- | --- | --- | --- |
| Antibiotic | No. of studies | Antibiotic resistance rate (95% CI) | n/N | Heterogeneity test, *I^2^* | Heterogeneity test, *P* value | Begg’s test | Egger’s test |
| Trimethoprim/ sulfamethoxazole | 23 | 22.3 (16.7-29.2) | 1118/4709 | 95.342 | 0.000 | 0.874 | 0.295 |
| Ticarcillin/ clavulanate | 19 | 38.8 (27.8-51) | 1418/3779 | 97.455 | 0.000 | 1.000 | 0.925 |
| Minocycline | 10 | 6 (2.6-13) | 138/1998 | 93.452 | 0.000 | 0.371 | 0.017 |
| Ceftazidime | 31 | 52.6 (44.5-60.6) | 2352/4745 | 95.766 | 0.000 | 0.634 | 0.579 |
| Ciprofloxacin | 14 | 47 (34.1-60.3) | 565/1401 | 93.805 | 0.000 | 0.661 | 0.192 |
| Levofloxacin | 20 | 22.5 (17.4-28.7) | 803/3644 | 92.873 | 0.000 | 0.346 | 0.335 |
| Chloramphenicol | 15 | 47.4 (32.2-63.1) | 1379/3195 | 98.122 | 0.000 | 0.552 | 0.280 |
| Tigecycline | 7 | 14.1 (7.7-24.3) | 277/2004 | 95.610 | 0.000 | 1.000 | 0.443 |
| Colistin | 3 | 67.1 (12.7-96.6) | 142/547 | 98.249 | 0.000 | 1.000 | 0.506 |
| High dose colistin | 1 | 14 (11.1-17.5) | 63/450 | 0.000 | 1.000 | - | - |
| Moxifloxacin | 3 | 15.7 (5.7-36.3) | 80/657 | 95.060 | 0.000 | 1.000 | 0.793 |
| Aztreonam | 6 | 80.5 (40.2-96.2) | 506/950 | 98.585 | 0.000 | 0.707 | 0.281 |
| Fosfomycine | 1 | 12.4 (9.7-15.8) | 56/450 | 0.000 | 1.000 | - | - |
| Cefoprazone/ Sulbactam | 6 | 30.4 (15.5-50.9) | 160/921 | 93.339 | 0.000 | 0.132 | 0.062 |
| Cefoprazone | 3 | 50.8 (23.6-77.5) | 179/466 | 96.340 | 0.000 | 1.000 | 0.258 |
| Cefepime | 7 | 46.8 (16.1-80.1) | 534/1107 | 98.538 | 0.000 | 1.000 | 0.916 |
| Piperacillin/ Tazobactam | 7 | 38.7 (14.3-70.5) | 487/851 | 97.714 | 0.000 | 0.763 | 0.052 |
| Meropenem | 8 | 92.7 (64.8-98.9) | 566/986 | 98.011 | 0.000 | 0.173 | 0.210 |
| Imipenem | 13 | 93.2 (76.1-98.3) | 764/884 | 93.598 | 0.000 | 0.854 | 0.261 |
| Doxycycline | 2 | 3.6 (2.2-5.8) | 32/900 | 50.755 | 0.154 | - | - |
| Ofloxacin | 1 | 20 (5-54.1) | 2/10 | 0.000 | 1.000 | - | - |
| Norfloxacin | 4 | 35.5 (27.4-44.4) | 43/122 | 0.000 | 0.524 | 0.308 | 0.520 |
| Amikacin | 11 | 77 (50.4-91.7) | 547/1095 | 97.245 | 0.000 | 0.697 | 0.259 |
| Gentamicin | 11 | 86.3 (73.8-93.4) | 384/467 | 86.393 | 0.000 | 0.391 | 0.057 |
| Tobramycin | 4 | 88.5 (80.6-93.4) | 110/122 | 33.551 | 0.211 | 0.734 | 0.821 |
| Netilmicin | 1 | 88 (75.8-94.5) | 44/50 | 0.000 | 1.000 | - | - |
| Ticarcillin | 3 | 30.3 (7-71.6) | 32/96 | 85.408 | 0.001 | 0.296 | 0.389 |
| Piperacillin | 5 | 54.8 (28.2-78.9) | 332/478 | 93.625 | 0.000 | 0.806 | 0.291 |
| Ampicillin | 2 | 88.6 (67.1-96.7) | 27/29 | 29.827 | 0.233 | - | - |
| Ampicillin/ sulbactam | 1 | 13.2 (9.7-17.6) | 37/281 | 0.000 | 1.000 | - | - |
| Cefoxitin | 1 | 35.3 (16.8-59.6) | 6/17 | 0.000 | 1.000 | - | - |
| Cefotaxime | 3 | 91.3 (80-96.5) | 53/57 | 0.000 | 0.654 | 0.296 | 0.256 |
| Ceftriaxone | 5 | 81.8 (18.7-98.9) | 135/344 | 96.884 | 0.000 | 1.000 | 0.312 |
| Cefuroxime | 2 | 94.1 (75-98.8) | 35/36 | 14.943 | 0.278 | - | - |
| Tetracycline | 2 | 93.9 (89-96.7) | 156/166 | 0.000 | 0.519 | - | - |
| Amoxicillin/ clavulanate | 2 | 51.5 (24.8-77.3) | 20/36 | 56.009 | 0.132 | - | - |

| Table4. Status of antibiotic-resistance among  *Stenotrophomonas maltophilia* in Regions of America | | | | | | | |
| --- | --- | --- | --- | --- | --- | --- | --- |
| Antibiotic | No. of studies | Antibiotic resistance rate (95% CI) | n/N | Heterogeneity test, *I^2^* | Heterogeneity test, *P* value | Begg’s test | Egger’s test |
| Trimethoprim/sulfamethoxazole | 20 | 13.1 (7.4-22.1) | 867/3173 | 96.8 | <.001 | 0.8 | 0.00009 |
| Ciprofloxacin | 20 | 58.4 (51.4-65.1) | 1381/2265 | 86 | 0.000 | 0.9 | 0.6 |
| Ceftazidime | 19 | 56.4 (47.8-64.6) | 1099/1799 | 90 | <.001 | 0.16 | 0.14 |
| Levofloxacin | 15 | 22.4 (16.5-29.7) | 374/1623 | 88 | <.001 | 0.3 | 0.14 |
| Imipenem | 11 | 94.9 (86.4-98.2) | 1168/1490 | 86 | 0.000 | 0.7 | 0.003 |
| Ticarcillin/clavulanate | 9 | 25.5 (14.2-41.4) | 437/1328 | 89.3 | 0.000 | 0.6 | 0.45 |
| Gentamicin | 8 | 78.3 (71.4-83.9) | 573/740 | 71 | 0.01 | 0.1 | 0.1 |
| Piperacillin-Tazobactam | 8 | 73.5 (56.9-85.4) | 384/533 | 90 | 0.000 | 0.7 | 0.3 |
| Chloramphenicol | 8 | 46.8 (34.8-59.1) | 895/1527 | 91 | <.001 | 0.26 | 0.08 |
| Ceftriaxone | 7 | 96.5 (93.7-98) | 482/491 | 42 | 0.1 | 0.7 | 0.04 |
| Amikacin | 7 | 79.2 (71.4-85.2) | 416/538 | 63 | 0.01 | 0.4 | 0.05 |
| Cefepime | 7 | 66.9 (50.9-79.7) | 382/616 | 91 | 0.000 | 0.7 | 0.3 |
| Meropenem | 6 | 95.6 (92.6-97.4) | 485/504 | 22 | 0.26 | 1.00000 | 0.8 |
| Tobramycin | 5 | 88.5 (70.9-96) | 205/234 | 82 | 0.000 | 0.2 | 0.2 |
| Aztreonam | 4 | 92.5 (88.2-95.3) | 231/247 | 33 | 0.2 | 0.7 | 0.4 |
| Ticarcillin | 4 | 74.6 (44.1-91.6) | 116/159 | 87 | 0.000 | 0.7 | 0.4 |
| Piperacillin | 4 | 68.9 (51.1-82.5) | 710/1078 | 84 | 0.000 | 1.00000 | 0.7 |
| Ofloxacin | 4 | 42.2 (18.6-70.1) | 130/354 | 94 | 0.000 | 1.00000 | 0.9 |
| Trovafloxacin | 4 | 28.6 (8.5-63.3) | 80/250 | 90 | 0.000 | 0.7 | 0.9 |
| Gatifloxacin | 4 | 18.8 (11.4-29.2) | 76/381 | 72 | 0.01 | 0.3 | 0.1 |
| Ampicillin | 3 | 97 (93.8-98.6) | 289/296 | 33 | 0.2 | 1.00000 | 0.4 |
| Cefotaxime | 3 | 92.4 (89.2-94.6) | 360/389 | 0.000 | 0.4 | 1.00000 | 0.6 |
| Moxifloxacin | 3 | 39.3 (8.2-82.3) | 43/95 | 93 | 0.000 | 1.00000 | 0.7 |
| Colistin | 3 | 25.5 (9-54.1) | 52/211 | 92 | 0.000 | 0.3 | 0.2 |
| Cefuroxime | 2 | 98.8 (94.4-99.8) | 129/130 | 0.000 | 0.8 | - | - |
| Polymyxin B | 2 | 69.8 (29.1-92.9) | 38/63 | 79 | 0.03 | - | - |
| Tigecycline | 2 | 5.5 (2.2-13) | 7/138 | 34 | 0.27 | - | - |
| Minocycline | 2 | 3 (1.1-7.6) | 4/133 | 0.000 | 0.68 | - | - |
| Fosfomycine | 2 | 38.5 (8.5-80.8) | 72/150 | 95 | 0.000 | - | - |

| Table5. Status of antibiotic-resistance among *Stenotrophomonas maltophilia* in European Region | | | | | | | |
| --- | --- | --- | --- | --- | --- | --- | --- |
| Antibiotic | No. of studies | Antibiotic resistance rate (95% CI) | n/N | Heterogeneity test, *I^2^* | Heterogeneity test, *P* value | Begg’s test | Egger’s test |
| Trimethoprim/ sulfamethoxazole | 26 | 20 (14.3-27.1) | 447/2403 | 90.937 | 0.000 | 0.186 | 0.802 |
| Ticarcillin/ clavulanate | 15 | 39.5 (28.9-51.2) | 417/952 | 88.806 | 0.000 | 0.113 | 0.246 |
| Minocycline | 4 | 6 (3.4-10.3) | 12/254 | 27.351 | 0.248 | 0.089 | 0.150 |
| Ceftazidime | 35 | 62 (54.4-69) | 1288/2083 | 87.914 | 0.000 | 0.656 | 0.911 |
| Ciprofloxacin | 42 | 54 (45.1-62.7) | 1897/3145 | 93.891 | 0.000 | 0.324 | 0.174 |
| Levofloxacin | 21 | 15.9 (10.7-23) | 375/2248 | 90.941 | 0.000 | 0.414 | 0.485 |
| Chloramphenicol | 3 | 50.2 (13.5-86.7) | 136/262 | 97.083 | 0.000 | 1.000 | 0.875 |
| Tigecycline | 5 | 13.2 (1.5-59.3) | 106/310 | 95.503 | 0.000 | 0.462 | 0.087 |
| Colistin | 7 | 52.7 (30-74.3) | 206/375 | 91.829 | 0.000 | 0.367 | 0.540 |
| High dose colistin | 2 | 8.1 (3.9-16) | 7/89 | 0.000 | 0.552 | - | - |
| Moxifloxacin | 5 | 13.4 (5.2-30.1) | 47/343 | 88.805 | 0.000 | 0.220 | 0.317 |
| Aztreonam | 10 | 86.2 (58.5-96.5) | 652/1290 | 96.005 | 0.000 | 1.000 | 0.842 |
| Fosfomycine | 3 | 33.4 (9.5-70.5) | 56/182 | 92.433 | 0.000 | 1.000 | 0.919 |
| Cefoprazone | 2 | 44.3 (2.9-95.5) | 53/261 | 97.498 | 0.000 | - | - |
| Cefepime | 13 | 69.9 (57.2-80.1) | 752/1080 | 92.074 | 0.000 | 0.222 | 0.628 |
| Piperacillin/ Tazobactam | 23 | 71.5 (59.6-81) | 1118/1489 | 92.005 | 0.000 | 1.000 | 0.857 |
| Meropenem | 19 | 92.7 (85.9-96.4) | 1124/1229 | 85.920 | 0.000 | 0.624 | 0.411 |
| Imipenem | 24 | 96.5 (92.4-98.4) | 1605/1736 | 87.949 | 0.000 | 0.003 | 0.001 |
| Doxycycline | 6 | 4.5 (2.7-7.2) | 16/393 | 0.000 | 0.648 | 1.000 | 0.427 |
| Ofloxacin | 7 | 27.3 (18.4-38.6) | 387/1204 | 91.389 | 0.000 | 0.229 | 0.175 |
| Norfloxacin | 4 | 82.9 (55.4-95) | 167/217 | 85.373 | 0.000 | 0.734 | 0.232 |
| Amikacin | 24 | 68 (58.3-76.4) | 1105/1598 | 89.172 | 0.000 | 0.413 | 0.855 |
| Gentamicin | 18 | 72.4 (60.2-82) | 1112/1500 | 92.028 | 0.000 | 0.939 | 0.799 |
| Tobramycin | 13 | 81.9 (71-89.4) | 874/1140 | 91.232 | 0.000 | 0.044 | 0.135 |
| Netilmicin | 7 | 70.5 (40-89.5) | 309/440 | 95.233 | 0.000 | 0.548 | 0.564 |
| Ticarcillin | 4 | 84.7 (64.9-94.3) | 402/461 | 90.183 | 0.000 | 0.734 | 0.878 |
| Piperacillin | 13 | 71.5 (53.4-84.6) | 887/1264 | 96.097 | 0.000 | 0.299 | 0.169 |
| Ampicillin/ sulbactam | 2 | 98 (86.9-99.7) | 55/55 | 0.000 | 0.571 | - | - |
| Cefotaxime | 8 | 88.9 (74-95.8) | 519/669 | 92.706 | 0.000 | 0.710 | 0.031 |
| Ceftriaxone | 8 | 88.8 (77.4-94.8) | 310/345 | 73.150 | 0.000 | 0.386 | 0.210 |
| Cefuroxime | 2 | 99.7 (97.9-100) | 355/355 | 0.000 | 0.743 | - | - |
| Tetracycline | 5 | 83.6 (45.8-96.9) | 182/247 | 94.079 | 0.000 | 0.806 | 0.217 |
| Amoxicillin/ clavulanate | 7 | 95.8 (74.6-99.4) | 488/528 | 92.414 | 0.000 | 0.133 | 0.232 |
